# Targeting Splenic Myeloid Cells with Nanobiologics to Prevent Postablative Pancreatic Cancer Recurrence via Inducing Antitumor Peripheral Trained Immunity

**DOI:** 10.1002/advs.202413562

**Published:** 2025-04-28

**Authors:** Shengbo Wu, Weichen Xu, Xuexia Shan, Liping Sun, Shuo Liu, Xixi Sun, Shaoyue Li, Xiaodong Hou, Xiaowan Bo, Chengzhong Peng, Bin Huang, Huixiong Xu, Wenwen Yue

**Affiliations:** ^1^ Ultrasound Department Zhejiang Hospital No. 1229 Gudun Road, Xihu District Hangzhou Zhejiang Province 310013 P. R. China; ^2^ Department of Medical Ultrasound Shanghai Tenth People's Hospital School of Medicine, Tongji University Shanghai 200072 P. R. China; ^3^ Shanghai Engineering Research Center of Ultrasound Diagnosis and Treatment National Clinical Research Center for Interventional Medicine Shanghai 200072 P. R. China; ^4^ Department of Ultrasound Zhongshan Hospital Institute of Ultrasound in Medicine and Engineering Fudan University Shanghai 200032 P. R. China

**Keywords:** irreversible electroporation, nanomaterials, pancreatic cancer, trained immunity

## Abstract

Minimally invasive irreversible electroporation ablation shows promise for pancreatic cancer (PCa), but the high recurrence and metastasis rates pose a therapeutic challenge for loco‐regional ablation treatment. Immunotherapy holds promise for preventing tumor recurrence, however, its efficacy against PCa remains limited. Here, using a preclinical model of PCa, it is identified that tumor development dramatically restructures the splenic immune landscape characterized by increased frequency of myeloid cells. Further, nanobiologics with high affinity for splenic myeloid cells using erythrocyte membrane fused with apoA1‐modified liposomes are presented. Biocompatible CaCO_3_ nanoparticles are incorporated to serve as a release reservoir of immunomodulatory therapeutics (muramyl dipeptide, MDP). The nanobiologics, MDCa@RBC‐Alipo, induce antitumor‐trained immunity by epigenetically and metabolically rewiring splenic myeloid cells, thereby overcoming the immunosuppressive tumor microenvironment in residual PCa following irreversible electroporation ablation. This approach enhances the therapeutic efficacy of aPD‐L1 and significantly inhibits tumor recurrence and hemorrhagic ascites development. The trafficking of MDP directly to the spleen highlights a previously uncharacterized pathway for inducing peripheral trained immunity, thereby presenting a novel therapeutic approach for locally advanced PCa treatment.

## Graphical abstract

Using a preclinical pancreatic cancer model, we identified that tumor progression restructures splenic immunity via myeloid cell expansion. MDCa@RBC‐Alipo nanobiologics were engineered to epigenetically and metabolically reprogram splenic myeloid cells, alleviating post‐ablation immunosuppression. This enhances the efficacy of aPD‐L1 therapy, suppresses tumor recurrence and hemorrhagic ascites, and reveals a splenic trained immunity pathway, providing a novel strategy for the advanced pancreatic cancer.

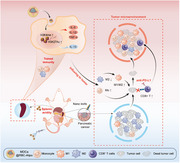

## 1 Introduction

Pancreatic cancer (PCa) is the third leading cause of cancer‐related death with a 5‐year survival rate of less than 7%.^[^ ^1^, ^2^ ^]^ At presentation, 75–80% of patients are not candidates for surgical resection due to the presence of metastatic or locally advanced disease.^[^ ^3^, ^4^ ^]^ The current established standard of care for advanced, unresectable PCa combines systemic chemotherapy and local radiotherapy, but with limited success.^[^ ^5^ ^]^ Therefore, a number of energy‐based ablative therapies have been examined as adjunct or stand‐alone therapy. Notably, irreversible electroporation (IRE), which utilizes high‐voltage electric fields to induce cell death through permanent membrane lysis, is the only ablative technique for advanced PCa approved by the FDA. It exhibits a remarkable ability to spare vital structures, such as major bile ducts and vessels, effectively mitigating the risk of complications including pancreatic fistula formation and bleeding.^[^ ^6^, ^7^ ^]^ Consequently, IRE has emerged as a superior therapeutic modality for locally advanced PCa supported by extensive global experience and international clinical guidelines.^[^ ^8^, ^9^ ^]^ In patients with PCa who respond well to IRE, most but not all tumor cells are eliminated, leaving behind a small fraction of residual tumor cells. Even after prolonged latency, these residual tumor cells can lead to recurrence, which remains a therapeutic dilemma for this loco‐regional ablation treatment.^[^ ^10^ ^]^

Over the last two decades, compelling clinical findings in immuno‐oncology have revolutionized cancer therapy. The success achieved in clinical trials with immunotherapy suggests its potential as a transformative approach for preventing tumor recurrence and metastasis.^[^ ^11^, ^12^ ^]^ However, its efficacy against PCa remains limited.^[^ ^13^, ^14^ ^]^ The majority of the advanced immunotherapies currently under development primarily rely on leveraging the adaptive immune system to induce or restore T cells'ability to mount an effective anti‐tumor response.^[^ ^15^ ^]^ While the value of these therapeutic approaches is indisputable, the utilization of self‐innate immune response in cancer therapy also holds great promise but is still largely unexplored.^[^ ^16^, ^17^, ^18^ ^]^

The innate immune system confers nonspecific defense against invading pathogens through myeloid cells with diverse functions, which can also invoke the subsequent antigen‐specific adaptive immune response. Myeloid cells can be activated by pathogen‐ or damage‐associated molecular patterns via pattern‐recognizing receptors.^[^ ^19^ ^]^ Upon their activation, these myeloid cells should undergo epigenetic and metabolic reprogramming, resulting in a hypersensitivity toward the subsequent encounters with both related and unrelated pathogens.^[^ ^20^, ^21^ ^]^ This phenomenon, known as “trained immunity”, can endure for several months. Therapeutically managing trained immunity represents a highly promising paradigm for cancer treatment.^[^ ^22^ ^]^ This can be regulated and maintained by inducing training properties in myeloid innate immune cells, including monocytes, macrophages, and dendritic cells.^[^ ^23^ ^]^ Thus, myeloid cell‐rich hematopoietic organs, such as the spleen and bone marrow, are important targets.^[^ ^24^, ^25^ ^]^

As the largest secondary lymphoid organ, the spleen functions as a site for storage and the rapid deployment of monocytes.^[^ ^26^ ^]^ Therefore, spleen targeting could elicit a potent and systemic immune response for effectively attacking tumor tissues. Also, precise therapeutic targeting of the spleen and myeloid cell subsets could take full advantage of the therapeutic potential of trained immunity and limit unwanted effects.^[^ ^27^, ^28^ ^]^ Toward this purpose, nanomaterials can be functionalized with trained immunity‐inducing molecular structures and designed to exhibit high spleen avidity, thereby facilitating their association with myeloid cells.^[^ ^29^ ^]^

Serving as a pivotal “filtration” organ in the human body, the spleen also functions as a crucial reservoir for blood. When blood is transported to the spleen, the aged or damaged red blood cells will be intercepted and then eliminated by phagocytes.^[^ ^30^ ^]^ Hence, the utilization of damaged erythrocyte membrane is suggested to hold potential for targeted spleen retention.^[^ ^31^ ^]^ Liposome‐based drug delivery system has been approved for clinical application.^[^ ^32^, ^33^ ^]^ Considering the poor drug‐loading efficiency of the erythrocyte membrane, a liposome comprising a phospholipid bilayer can be employed for fusion with the erythrocyte membrane.^[^ ^34^ ^]^ The fusion drug carrier should not only retain the excellent biocompatibility and spleen‐targeting ability of the erythrocyte membrane but also overcome the drug loading defect.^[^ ^35^, ^36^ ^]^ We foresee that if appropriately designed, such nanomaterial‐mediated trained immunity‐promoting therapy may elicit a durable anti‐cancer innate immune response by stimulating the generation of trained myeloid cells in the spleen and their subsequently infiltration into the tumor microenvironment (TME). Concurrently, these trained cells mobilize adaptive immune responses via enhanced T‐cell activation while increasing the immune system's susceptibility to checkpoint‐inhibitor drugs.

**Figure 1:**
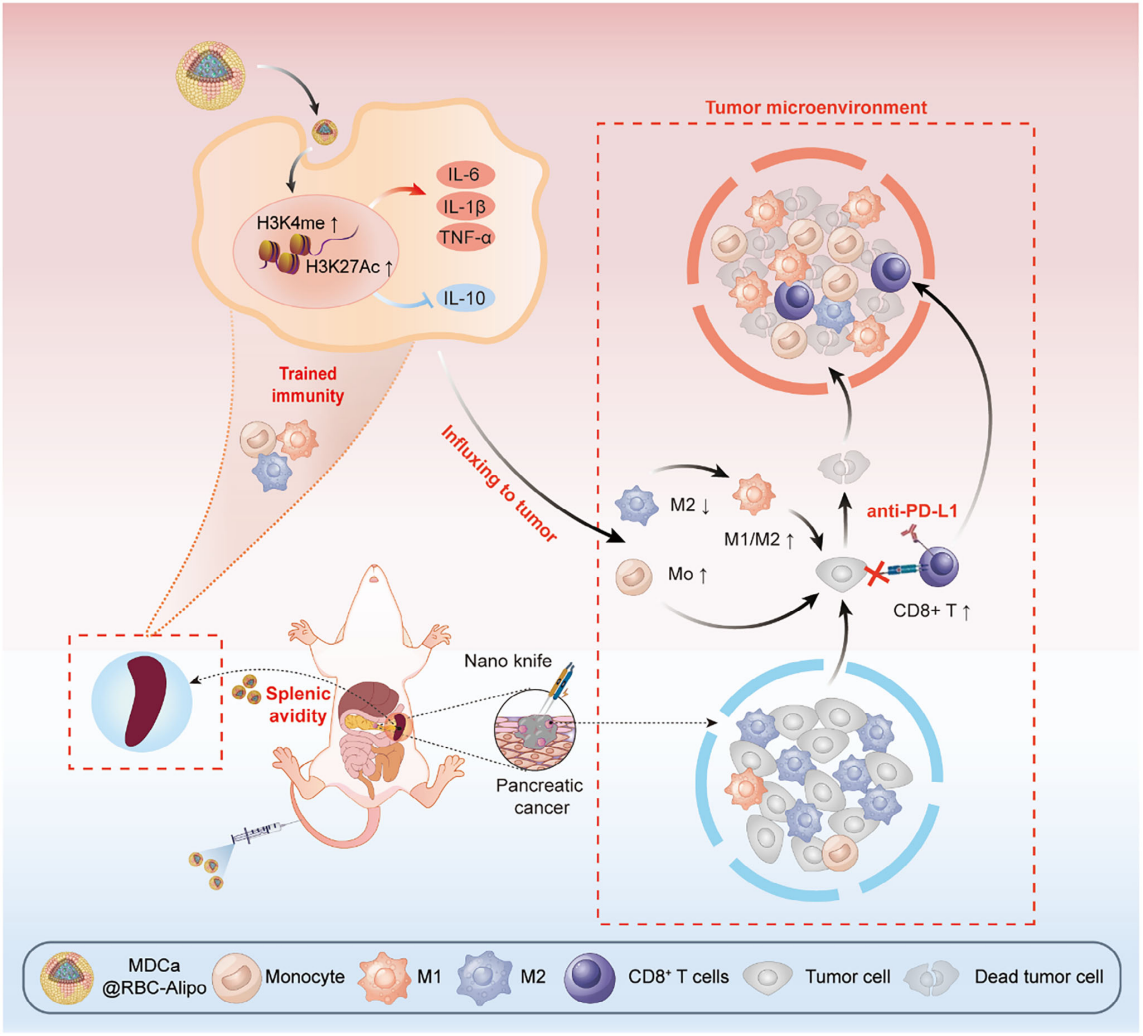
Schematic illustration of targeting splenic myeloid cells with nanobiologics to prevent postablative pancreatic cancer recurrence via inducing antitumor peripheral trained immunity. After administration, the nanobiologics, MDCa@RBC‐Alipo, can effectively accumulate in the spleen and establish favorable interactions with myeloid cells, thereby inducing antitumor peripheral trained immunity through epigenetic and metabolic rewiring of splenic myeloid cells. This approach further overcomes the immunosuppressive tumor microenvironment following irreversible electroporation ablation treatment and enhances the therapeutic effects of aPD‐L1 blockade therapy. Consequently, it significantly inhibits postablative pancreatic tumor recurrence and the development of hemorrhagic ascites while prolonging the survival of immunocompetent mice bearing well‐established orthotopic pancreatic tumors. Mo, Monocyte; M1, M1‐like macrophage; M2, M2‐like macrophage.

Here, we describe an experimental PCa model system that links malignant tumor outgrowth and its subsequent biological response to the reorganization in the splenic immune cells. Specifically, we observe that tumor development dramatically restructures the immune landscape in the spleen characterized by the increased frequencies of myeloid cells, including monocytes and macrophages along with reductions in B cell and cytotoxic T‐lymphocyte populations. Such cancer‐induced alterations in the splenic immune population are believed to be adaptations to the heightened demand for myeloid cells by tumors, which can subsequently traffic to the TME and contribute to the local immunosuppression. Based on these analyses, we further present the development and therapeutic application of a novel nanobiologic platform with high affinity for splenic myeloid cells using erythrocyte membrane fused with apolipoprotein A‐1 (apoA1)‐modified liposomes. Biocompatible CaCO_3_ nanoparticles are incorporated into the fusion erythrocyte membrane to serve as a release reservoir of immunomodulatory therapeutics (muramyl dipeptide, MDP). The nanobiologics, MDCa@RBC‐Alipo, can effectively accumulate in the spleen and establish positive interactions with myeloid cells, thereby inducing antitumor immunity through epigenetic and metabolic rewiring of splenic myeloid cells. Our findings demonstrated that the MDCa@RBC‐Alipo‐mediated peripheral trained immunity effectively reversed the immunosuppressive TME in residual PCa following IRE therapy, thereby augmenting aPD‐L1‐associated antitumor responses. This resulted in significant suppression of tumor growth and hemorrhagic ascites formation, ultimately leading to prolonged survival in immunocompetent mice harboring well‐established orthotopic pancreatic tumors (**Figure** **1**). The direct trafficking of MDP to the spleen highlights a previously uncharacterized pathway for the induction of peripheral trained immunity. The peripheral‐trained immunity‐promoting therapy offers a novel strategy for the treatment of locally advanced PCa. Our findings hold significant relevance for advancing the field of cancer immunotherapy and improving clinical outcomes for patients with this challenging disease.

## 2 Results

### 2.1 Tumor Burden Restructures the Immune Landscape in Spleen

**Figure 2:**
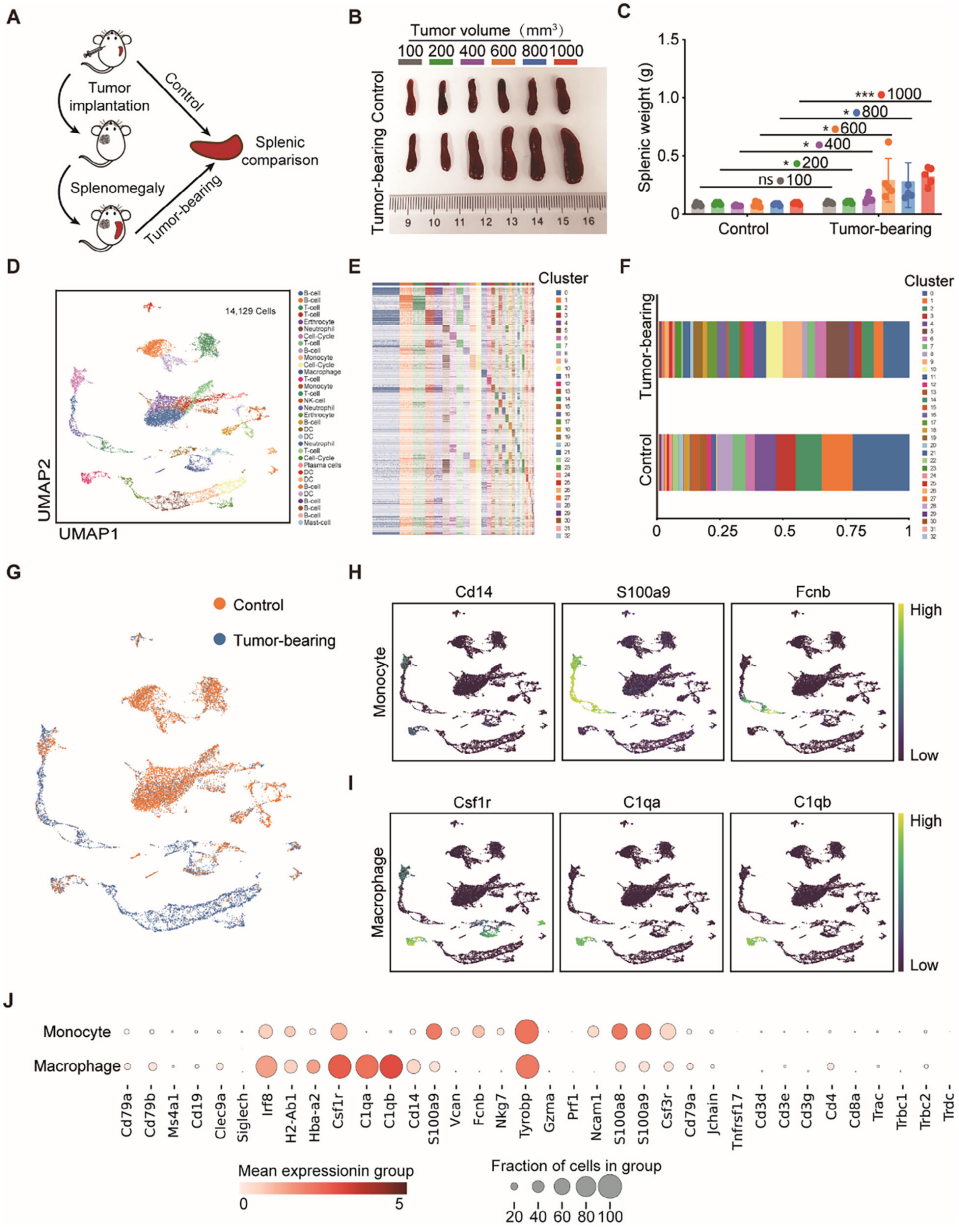
Tumor burden restructures the immune landscape in the spleen. A) Schematic illustration of splenic alterations in tumor‐bearing mice. B) Representative splenic photographs depicting mice with varying tumor volumes (100–1000 mm^3^). C) Splenic weight in mice bearing tumors of varying volumes (100–1000 mm^3^) (*n* = 4–5 mice). D) 2D UMAP representation of 14 129 cells aggregated from the two experimental samples, resulting in 33 distinct clusters. E) Heatmap illustrating the expression patterns of aggregated marker genes across all clusters. F) Bar graphs showing the relative frequency of cells in each cluster across samples. G) Revealing UMAP dimension reduction of control and tumor‐bearing samples individually. Distribution of single‐cell expression patterns in cell populations recognized in the phenotypic classification of monocytes (H) and macrophage (I). J) A dot plot was generated to display the top 36 enriched genes in monocyte and macrophage phenotyping, illustrating both the average expression level and percentage of cells expressing select genes.

To describe the cancer‐associated changes in the spleen, we first established a murine PCa model by subcutaneously injecting murine Pan02 cells into the C57/BL6 mice and then examined the length and weight of mouse spleens at different durations of tumor‐bearing (**Figure** **2**A,B). We found that the spleen of tumor‐bearing hosts significantly enlarged as the tumor progressed (≥400 mm^3^), particularly during later stages, when compared to controls (Figure 2C; Figure S1, Supporting Information).

To elucidate the mechanism underlying this tumor outgrowth‐induced splenic response, single‐cell RNA sequencing (scRNA‐Seq) analysis was performed on the mouse spleens with a tumor volume of 400 mm^3^ and control samples. A total of 14129 cells from two samples were aggregated and visualized in a 2D UMAP representation, where 33 distinct clusters were identified using the Louvain algorithm partitioning and k‐nearest neighbors approach (Figure 2D,E). The relative frequency of each cluster within the tumor‐bearing experimental group and control group was evaluated (Figure 2F). According to the high expression levels of genes, clusters 9 and 13 were identified as monocytes based on their elevated expression of CD14, S100A9, and FCNB. Meanwhile, cluster 11, which exhibited preferential enrichment of CSF1R, C1QA, and C1QB, was designated as macrophages (Figure 2G–J). Increased CD14‐labeled monocytes in the spleen of tumor‐bearing mice may activate the therapeutic potential of innate immunity through the TLR4 signaling pathway (Figure 2H). Macrophage C1q definition could be activated by MDP pathogen‐associated molecular patterns, enhancing the recognition of tumor cells and macrophage phagocytosis (Figure 2I). The pancreatic carcinoma model exhibited extensive remodeling of the splenic immune population, characterized by phenotypic shifts and increased frequencies of myeloid cells (i.e., monocytes and macrophages) in clusters 9, 11, and 13, accompanied by reductions in B cell and T cell populations (Figure 2F). In support, multiple immunohistochemistry (mIHC) analysis of splenic tissue in orthotopic pancreatic tumor mice also revealed a significant increase in CD11b^+^ myeloid cell populations concomitant with a marked reduction in both CD3^+^ T cells and CD19^+^ B cells compared to the control group (Figure S2, Supporting Information). This immune cell redistribution pattern suggests tumor‐mediated systemic immunosuppression characterized by myeloid expansion and lymphoid depletion within peripheral splenic immunity.

Further investigation into the heterogeneity of myeloid cells revealed that clusters C9, 11, and 13 exhibited elevated expression levels of myeloid‐derived suppressor cell (MDSC)‐associated genes, such as S100A8 and S10019, which have previously been utilized for defining MDSCs in cancer models^[^ ^37^, ^38^ ^]^ (Figure S3A, Supporting Information). TAMs were polarized to a pro‐tumorigenic M2‐phenotype, expressing the CSF1R, which was observed at a higher frequency in splenic macrophages in tumor‐bearing mice (Figure 2I). The gene signature of MDSC‐like cells was subsequently subjected to Gene Ontology (GO) term analysis (Figure S3B, Supporting Information). It was revealed that genes enriched in the biological process (BP) of positive regulation of the immune effector process, antigen processing and presentation of peptide antigen, and lymphocyte‐mediated immunity exhibited significant down‐regulation. As the previous research, the main characteristic that defines MDSCs is their ability to inhibit immune responses mediated by T cells and B cells, and ultimately promote the development of immunosuppression in the peripheral immune system. The enriched GO analysis of the respiratory electron transport chain showed that metabolic reprogramming occurred in splenic myeloid cells, thereby corroborating the aforementioned findings.^[^ ^38^ ^]^ However, the upregulated enrichment primarily pertained to innate immune responses, specifically associated with chemokine production, leukocyte aggregation, and leukocyte migration involved in inflammatory responses. This suggests that peripheral myeloid cells may possess the capability to respond to recruitment signals originating from sites of inflammation such as the primary tumor or metastatic sites. Meanwhile, cluster 13 exhibited a high enrich of chromosome separation, indicating the existence of a proliferative pool of myeloid cells in the spleen. Therefore, the targeting of splenic myeloid cells for differentiation into pro‐inflammatory macrophages presents significant potential in eliciting a robust and systemic immune response that effectively targets tumor tissues.

### 2.2 Developing and Characterizing Nanobiologics with High Affinity for Splenic Myeloid Cells

Efficient nanotherapeutic modulation of the innate immune system requires a biocompatible nanocarrier exhibiting high affinity toward both the spleen and myeloid cells. High‐density lipoprotein (HDL) serves as a natural nanocarrier responsible for the transportation of fat and other biomolecules.^[^ ^39^ ^]^ ApoA1 is the predominant protein constituent of native HDL and interacts with scavenger receptors and the adenosine triphosphate (ATP)–binding cassette transporters ABCA1 and ABCG1, abundantly present on myeloid cells.^[^ ^40^ ^]^ Working as the important “filter” in the body, damaged red blood cells will be intercepted by phagocytes in the spleen. Therefore, the damaged erythrocyte membrane has the potential for “targeting” retention in the spleen.^[^ ^41^ ^]^ On the basis of apoA1 and fusion erythrocyte membrane, we developed nanobiologics (MDCa@RBC‐Alipo) that exhibit a remarkable affinity toward both spleen and myeloid cells.

**Figure 3:**
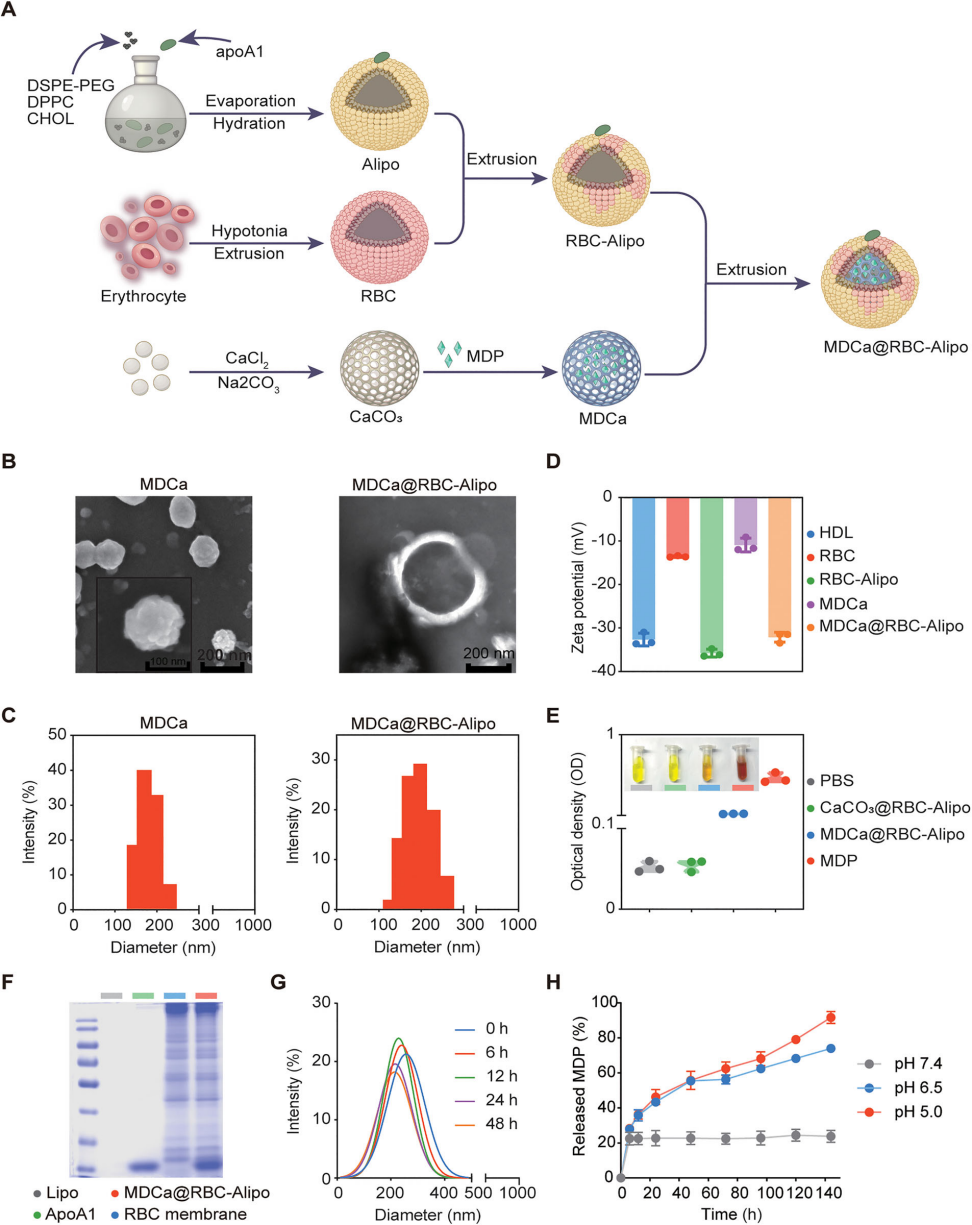
Schematic and characterization of MDCa@RBC‐Alipo nanobiologics. A) A scheme indicating the step‐by‐step synthesis of MDCa@RBC‐Alipo. B) Representative transmission electron microscopy image of MDCa and MDCa@RBC‐Alipo nanoparticles. C) Particle‐size distribution of MDCa and MDCa@RBC‐Alipo measured by dynamic light scattering. D) Zeta potential variations in the preparation procedure of MDCa@RBC‐Alipo nanoparticles. Data were presented as means ± SD (*n* = 3). E) Drug loading ratio and the relative digital photo (inset) of MDP. Data were presented as means ± SD (*n* = 3). F) SDS‐PAGE pattern of proteins from liposome, apoA1, red blood cell membrane, and MDCa@RBC‐Alipo lysates. G) Size changes of MDCa@RBC‐Alipo nanoparticles in PBS to prove stability. H) Cumulative release profiles of MDP from CaCO_3_ in solutions at different pH values. Data were presented as means ± SD (*n *= 3).

The MDCa@RBC‐Alipo was synthesized as illustrated in **Figure** **3**A. Briefly, MDCa nanoparticles with an encapsulation efficiency of ≈64% were first fabricated by precipitation of Ca^2+^ and CO_3_^2−^ in a solution containing poly (ethylene glycol)‐bpoly (glutamic acid) (PEG‐b‐P(Glu)) block copolymers. Erythrocyte membrane was used to decorate apoA1‐modified liposomes (RBC‐Alipo) and then loaded with MDCa to yield the final MDCa@RBC‐Alipo nanobiologics for further experiments.

Transmission electron microscope (TEM) images clearly displayed the spherical morphology of MDCa, and the MDCa@RBC‐Alipo nanobiologics were well dispersed in aqueous solution and appeared as quasi‐spheres under TEM observation (Figure 3B). The hydrodynamic diameter of the MDCa nanoparticle increased from 180 to 200 nm upon being coated with RBC‐Alipo (Figure 3C). The successful construction of the fusion erythrocyte membrane and the encapsulation of MDCa was proved by the shifting zeta potential during the stepwise synthesis (Figure 3D) and also validated by the changes in colorimetric assay (Figure 3E). SDS‐polyacrylamide gel electrophoresis (SDS‐PAGE) verified that the MDCa@RBC‐Alipo nanobiologics had similar protein expression with apoA1 protein and erythrocyte membrane (Figure 3F). These data demonstrated that the fusion erythrocyte membrane was successfully loaded into the MDCa nanoparticles and apoA1 was successfully bound on the surface of MDCa@RBC‐Alipo.

The MDCa@RBC‐Alipo exhibited minimal size variation over a 48 h period in phosphate‐buffered saline (PBS) containing 5 mg mL^−1^ bovine serum albumin, proving the stability of nanobiologics in the internal environments (Figure 3G). As reported, the calcium‐carbonate liposome would be internalized by the endo/lysosomal pathway.^[^ ^42^ ^]^ Thus, we evaluated the MDP release from MDCa@RBC‐Alipo in PBS at pH 6.5 and 5.0, simulating the conditions found in endosomes and lysosomes, respectively. In pH of 6.5 and 5.0, the MDCa@RBC‐Alipo released 73.5% and 91.7% of MDP in 144 h. Whereas, in pH of 7.4, only 23.8% of MDP was released from the MDCa@RBC‐Alipo in 144 h (Figure 3H). The data suggest that the nanoparticles could enhance the stability of MDP in blood and enable pH‐triggered release, facilitating drug delivery from endo/lysosomes into the cytoplasm.

### 2.3 Trained Immunity Induced by MDCa@RBC‐Alipo Nanobiologics

**Figure 4:**
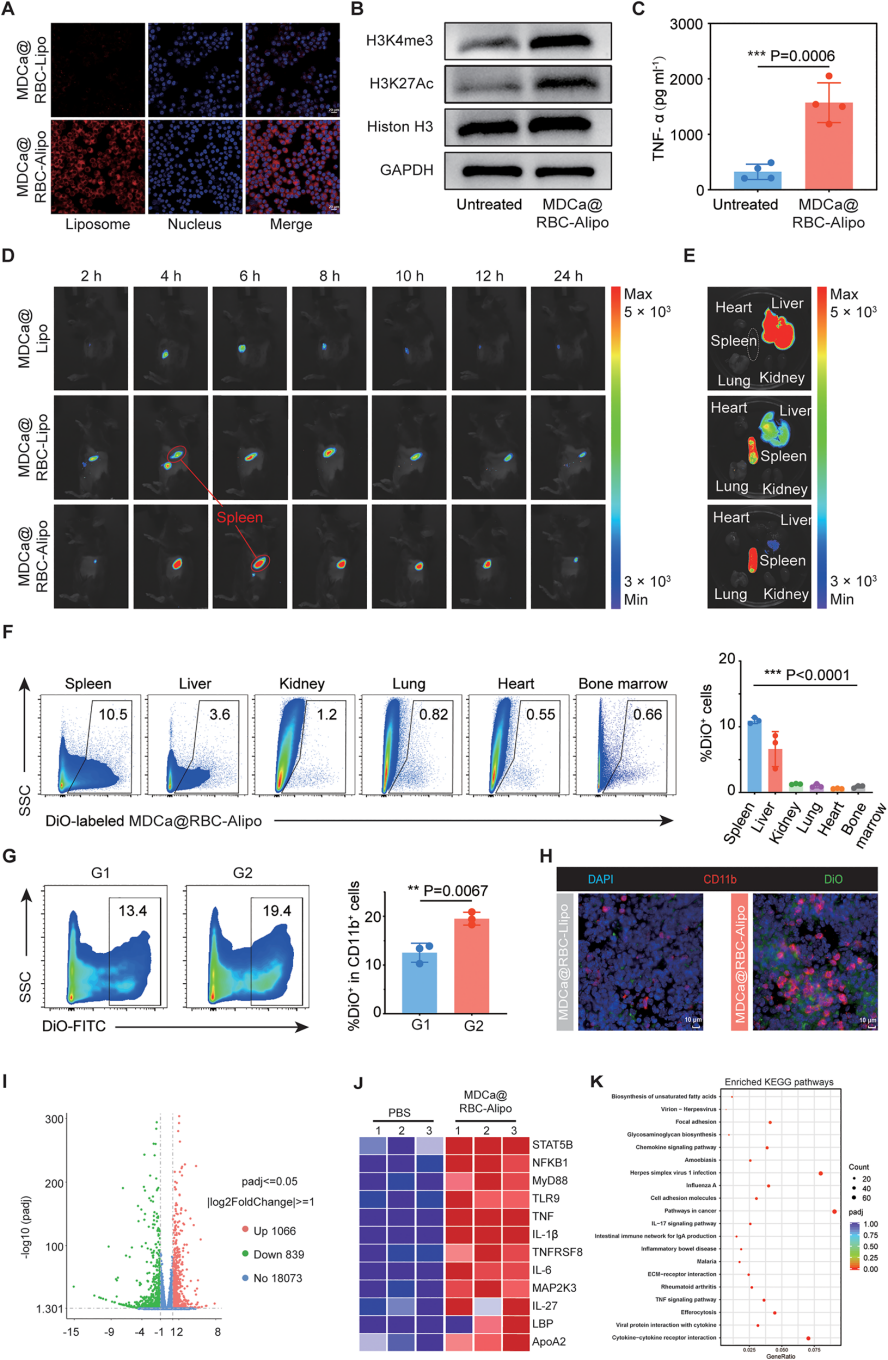
MDCa@RBC‐Alipo provokes trained immunity activation via targeting myeloid cells in the spleen. A) Representative confocal laser scanning microscopy of endocytosis‐dependent uptake of nanoparticles (Cy5.5‐DSPE‐PEG) in J774A.1 cells loaded with or without apoA1. Scale bars, 20 µm (*n* = 3). B) Histones of J774A.1 cells preincubated with PBS or MDCa@RBC‐Alipo for 24 h were isolated and subjected to western blot analysis (*n* = 3). C) Seven days after preincubated with PBS or MDCa@RBC‐Alipo, cells were restimulated with LPS for 24 h. TNF‐α was measured using ELISA (*n* = 4). D) Fluorescence images to reveal the biodistribution of MDCa@Lipo, MDCa@RBC‐Lipo, or MDCa@RBC‐Alipo post i.v. injection at the indicated time points. E) Fluorescence imaging of dissected organs 6 h after intravenous injection of MDCa@Lipo, MDCa@RBC‐Lipo, or MDCa@RBC‐Alipo. F) Representative flow‐cytometry plots and quantification of the biodistribution of DiO‐MDCa@RBC‐Alipo in vital organs 6 h after administration (*n* = 3). G,H) The affinity of apoA1‐loaded nanoparticles to splenic myeloid cells was determined by flow cytometry (G) and mIHC (H). I) After a seven‐day period following intravenous injection of MDCa@RBC‐Alipo or PBS, splenic CD45^+^CD11b^+^ populations were sorted and subjected to RNA‐Seq analysis (*n* = 3). The distribution of p values (−log10(*p*‐value)) and fold changes (log2 FC) of differentially expressed genes are represented. J) Heatmap of differentially expressed genes associated with proinflammatory and trained immunity (*n *= 3). Red and blue colors represent upregulated or downregulated genes, respectively. K) KEGG enriched dot of differentially expressed genes. Statistical difference was calculated using a two‐tailed unpaired student's *t*‐test. Data were expressed as means ± SD. *P < 0.05, **P < 0.01, and ***P < 0.001.

After determining the physicochemical characteristics of MDCa@RBC‐Alipo nanobiologics, we extensively studied their properties to induce trained immunity on mononuclear macrophages. Initially, we conducted an investigation into the biosafety and targeting properties of the MDCa@RBC‐Alipo nanobiologics under intracellular conditions. The cytotoxicity assays demonstrated negligible toxicity of MDCa@RBC‐Alipo toward J744A.1 cells and Pan02 cells, even at doses of MDP up to 30 ppm (Figure S4, Supporting Information). Afterward, colocalization of fluorescent signals was observed after co‐incubation with J774A.1 cells for 24 h. Compared with liposomes, apoA1‐modified liposomes were easier to be absorbed by macrophages and so was the fusion vector RBC‐Alipo and MDCa@RBC‐Alipo demonstrating the high targeting efficacy (**Figure** **4**A; Figure S5, Supporting Information).

Then, cells were analyzed for epigenetic changes using western blot analysis, and the medium was subjected to the multiplex cytokine analysis after treatment with MDCa@RBC‐Alipo. As shown in Figure 4B, the expression levels of H3K27Ac and H3K4me3 were significantly enhanced in MDCa@RBC‐Alipo‐trained J774A.1 cells (Figure 4B; Figure S6, Supporting Information). Several inflammatory cytokines (i.e., TNF‐α, IL‐6, and IL1β) have been used as surrogate biomarkers to evaluate the trained immunity. Compared to the PBS group, cells trained with MDCa@RBC‐Alipo exhibited significantly higher secretions of TNF‐α, IL‐6, and IL1β, as well as lower levels of IL‐10 according to ELISA quantitative assay (Figure 4C; Figure S7, Supporting Information). Since trained immunity is also associated with metabolic reprogramming, we quantified lactate levels to investigate the potential involvement of glycolysis. Indeed, MDCa@RBC‐Alipo‐trained J774A.1 cells secreted more lactate compared to untrained cells (Figure S8, Supporting Information), further corroborating that our nanobiologics effectively induce cellular training in vitro.

Encouraged by the in vitro results, MDCa@RBC‐Alipo nanobiologics were further employed for the in vivo evaluation of trained immunity. Before we ventured into therapeutic studies, the biodistribution of MDCa@RBC‐Alipo in mice was evaluated by tracking the fluorescence of Cy5.5‐labeled nanobiologics using an in vivo fluorescence imaging system (Figure 4D). Compared with the MDCa@Lipo group, with the fusion of erythrocyte membrane, the MDCa@RBC‐Lipo and MDCa@RBC‐Alipo nanobiologics emerged with obvious accumulation in the spleens 4 h after intravenous injection, and the fluorescence signal can be continuously monitored within 24 h (Figure S9, Supporting Information). The dissected spleens in the MDCa@RBC‐Alipo group exhibited significantly higher fluorescence intensity 6 h after injection via the tail vein (Figure 4E; Figure S10, Supporting Information). Simultaneously, a minor fluorescent signal was also observed in the liver, which serves as the primary metabolic pathway for the nanobiologics. However, in contrast to the limited diffusion and rapid clearance observed with other groups, the nanobiologics exhibited enhanced spleen penetration, widespread distribution throughout the entire spleen, and prolonged retention. In addition, after six hours of i.v. injection of DiO‐MDCa@RBC‐Alipo, the spleen, liver, kidney, and lung were harvested for subsequent assessment of the presence of DiO‐MDCa@RBC‐Alipo in each organ using flow cytometry (FCM). The spleen exhibited a remarkable and anticipated presence of the MDCa@RBC‐Alipo (Figure 4F). Especially, the utilization of FCM and mIHC techniques demonstrated the presence of DiO‐labeled organs in mice injected with MDCa@RBC‐Lipo and MDCa@RBC‐Alipo, providing supporting evidence for the strong affinity of ApoA1 toward splenic myeloid cells(Figure 4G,H). As demonstrated in Figure 4F, DiO‐MDCa@RBC‐Alipo nanoparticles exhibited negligible accumulation in the bone marrow. This observation supports the hypothesis that these nanoparticles preferentially induce trained immunity in peripheral splenic myeloid cells rather than mobilizing myeloid progenitors in the bone marrow niche.

After discovering that MDCa@RBC‐Alipo displays tropism toward the splenic myeloid cells, we further investigate whether these cells displayed a phenotype of trained immunity following treatment with MDCa@RBC‐Alipo. To achieve this, we employed a standard training protocol in which mice were treated with MDCa@RBC‐Alipo, MDP, or PBS. And 7 days later the myeloid cells in the spleen were sorted and restimulated with LPS. We subsequently evaluated the histone modifications of the cells and quantified the levels of TNF‐α, IL‐6, and IL1β in the supernatants. Western blot analysis revealed that both the MDCa@RBC‐Alipo group and MDP group exhibited elevated expression levels of H3K27Ac and H3K4me3 (Figure S11, Supporting Information). Also, as compared to cells from the PBS group, cells from the MDCa@RBC‐Alipo trained mice that were restimulated with LPS produced significantly more TNF‐α, IL‐6, and IL1β, indicating a pronounced training effect on splenic myeloid cells (Figure S12, Supporting Information). Moreover, MDCa@RBC‐Alipo nanobiologics demonstrated superior efficacy in activating trained immunity compared to free MDP.

To further explore the underlying mechanism of the promoted trained immunity effects of MDCa@RBC‐Alipo, we conducted RNA‐seq analysis for the splenic myeloid cells (CD45^+^CD11b^+^ cells) after MDCa@RBC‐Alipo treatment as well as untreated controls. The analysis revealed a total of 1950 differentially expressed genes (DEGs), with 1066 genes upregulated and 839 genes downregulated in the MDCa@RBC‐Alipo‐trained setting (Figure 4I). Gene set enrichment analyses (GSEA) further confirmed the previously investigated upregulations in pathways related to TNF‐α and cytokine production (Figure S13A, Supporting Information). The MDCa@RBC‐Alipo group exhibited significant enrichment of genes associated with leukocyte chemotaxis and reactive oxygen species, indicating enhanced mobility and lethality of splenic myeloid cells (Figure S13A, Supporting Information). Notably, genes encoding TNF‐α, proinflammatory cytokines, and chemokines were significantly up‐regulated in trained myeloid cells (Figure 4J). As expected, the enrichment of KEGG pathways was found to be associated with the TNF signaling pathway, chemokine signaling pathway, and efferocytosis, providing further evidence that supports the involvement of trained immunity in this process (Figure 4K; Figure S13B, Supporting Information). Collectively, the in vivo biodistribution and the trained immunity assays revealed the favorable characteristics of the MDCa@RBC‐Alipo nanobiologics for immunotherapeutic studies.

### 2.4 MDCa@RBC‐Alipo Nanobiologics for Inhibiting PCa Recurrence after IRE Treatment

**Figure 5:**
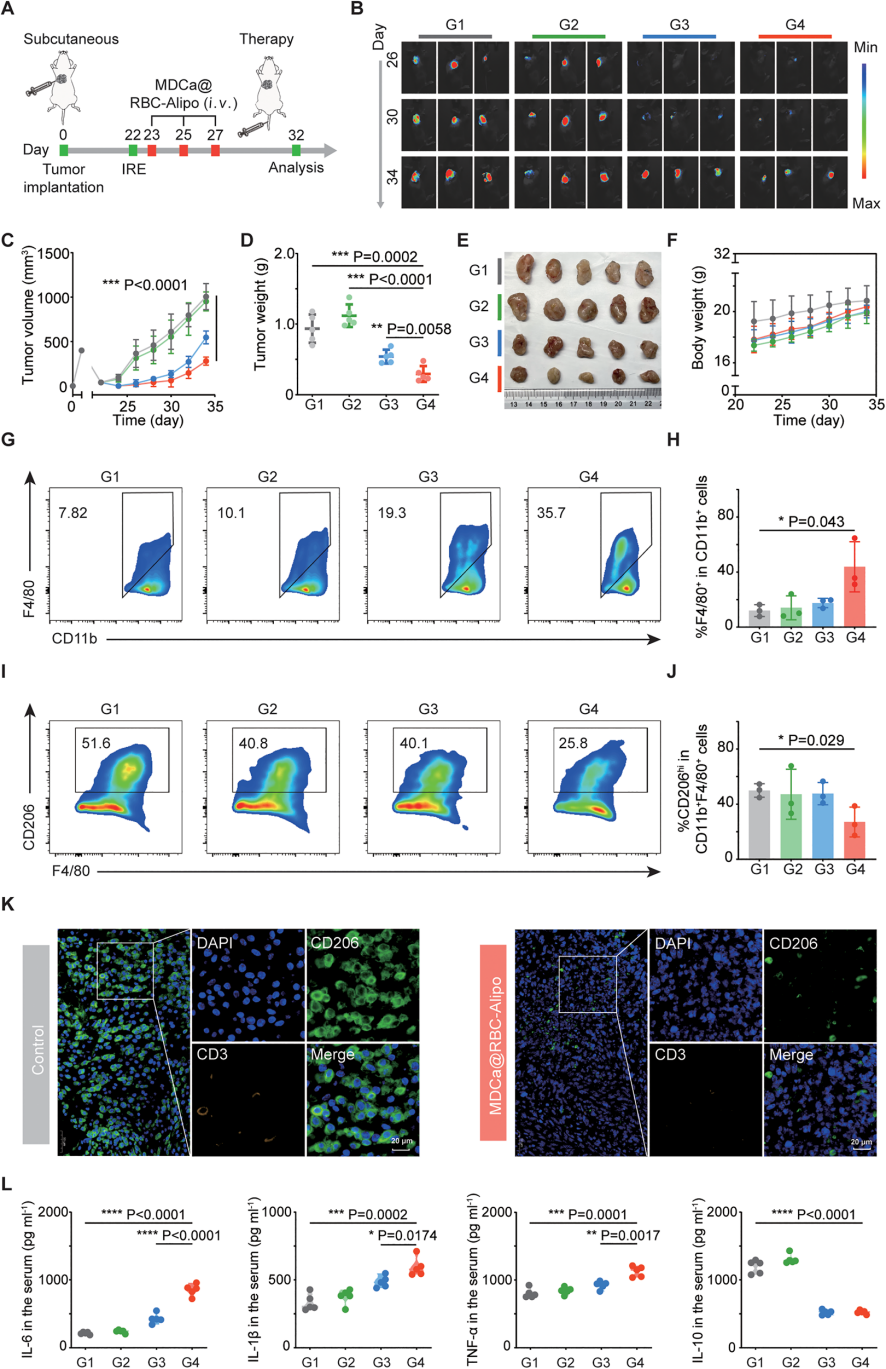
MDCa@RBC‐Alipo for inhibiting recurrence of PCa after irreversible electroporation treatment. A) Schematic illustration of the experiment design to assess the in vivo MDCa@RBC‐Alipo‐based peripheral trained immunity and its triggered immune responses. IRE, irreversible electroporation. B) Representative bioluminescence images of Luc+Pan02 tumor after various treatments as indicated. C) Average tumor growth curves after various treatments (*n* = 5). D) Average weights of tumors at the end of treatment (*n* = 5). E) Digital photo of tumors at the end of treatment. F) Time‐dependent body weight surveillance of mice after different treatments (*n* = 5). G–J) Representative flow cytometric analysis and relative quantification of macrophages (F4/80^+^CD11b^+^CD45^+^) and TAM‐M2 (CD206^hi^CD11b^+^F4/80^+^CD45^+^) in tumor. K) Polychromatic immunofluorescent staining images of tumors showing DAPI (blue), CD206^+^ (green), and CD3^+^ (orange) cell infiltration for control and MDCa@RBC‐Alipo groups. L) Cytokine levels of IL‐6, IL‐1β, TNF‐a, and IL‐10 in the serum after various treatments (*n* = 5). G1, Control; G2, RBC‐Alipo; G3, MDCa; G4, MDCa@RBC‐Alipo. Statistical difference was calculated using a two‐tailed unpaired student's *t*‐test. Data were expressed as means ± SD. *P < 0.05, **P < 0.01 and ***P < 0.001.

It has been reported that clinically used IRE commonly showed limited therapeutic efficacy toward tumors as the result of incomplete ablation. Herein, we carefully investigated the potency of our MDCa@RBC‐Alipo nanobiologics in augmenting the therapeutic effectiveness of IRE by utilizing their capacity in inducing trained immunity (**Figure** **5**A). On day 22 after inoculation with Luc+ Pan02 cells, the visible tumor was partially removed with the IRE procedure by using a commercial IRE system. Then, the C57BL/6 mice bearing residual tumors were randomly divided into four groups (*n* = 5) before each treatment, including the Control group (PBS), RBC‐Alipo group, MDCa group, and MDCa@RBC‐Alipo group (dose of MDP = 1.5 mg kg^−1^). The mice were intravenously injected with PBS/RBC‐Alipo/MDCa/MDCa@RBC‐Alipo according to groups on days 23, 25, and 27. The tumor growth of mice was monitored by capturing bioluminescence signals from Pan02 cells. Bioluminescence images showed no appreciable suppressive effect of RBC‐Alip on tumor growth (Figure 5B). We observed that the MDCa slowed the growth of residual tumors to some extent compared to the PBS group. Of note, mice receiving MDCa@RBC‐Alipo treatment showed a significant delay of tumor growth (Figure 5C–E). The variation trend of tumor volume was consistent with that of tumor weight (Figure 5D). As indicated in Figure 5D, the MDCa@RBC‐Alipo treatment acquired the largest reduction of tumor weight, which should be attributed to the high targeting efficacy of the fusion vector. The average body weight of mice was not obviously affected after receiving MDCa@RBC‐Alipo nanobiologics treatment (Figure 5F). Also, the serum biochemistry assay and the histology analysis of major organs obtained from mice 10 days after treatment indicated no obvious toxic‐effects induced by this immunotherapeutic strategy (Figure S14, Supporting Information).

### 2.5 Mechanism of the Antitumor Immunity Elicited by MDCa@RBC‐Alipo Nanobiologics

We next investigated whether MDCa@RBC‐Alipo‐mediated trained innate immune responses in the spleen would have a certain effect on the aggravated immunosuppressive phenotype of residual tumors following inadequate ablation. Tumor tissues were collected and analyzed by FCM and mIHC on the tenth day after different treatments. The flow cytometry analysis revealed that MDCa@RBC‐Alipo nanobiologics effectively enhanced the frequency of tumor‐infiltrating CD45^+^CD11b^+^F4/80^+^ macrophages compared with other controls (Figure 5G,H). We further subdivided these macrophages into M1‐like macrophages (CD11b^+^F4/80^+^CD86^hi^, TAMs‐M1) and M2‐like macrophages (CD11b^+^F4/80^+^CD206^hi^, TAMs‐M2). Although TAMs‐M1 was not significantly affected, we observed a significant reduction of TAMs‐M2 in the MDCa@RBC‐Alipo‐treated mice (Figure 5I,J). The ratio of M1/M2 was detected to be the highest in the MDCa@RBC‐Alipo group (Figure S15, Supporting Information), consistent with the obtained strongest antitumor effects in this group. Notably, neither CD4^+^ nor CD8^+^ T‐cells exhibited an increase, further supporting that the reduction in tumor burden was driven by the MDCa@RBC‐Alipo‐induced innate antitumor immunity (Figure S16, Supporting Information). In support, mIHC also confirmed the above results (Figure 5K), and the tumor exhibited an elevated population of CD11b^+^ myeloid cells, which demonstrated enhanced production of TNF‐α as a result of exposure to MDCa@RBC‐Alipo (Figure S17, Supporting Information). Likewise, in the MDCa@RBC‐Alipo treatment group, significantly elevated levels of serum cytokines such as TNF‐α, IL‐6, and IL1‐β, which play crucial roles in cellular immunity against cancer, were observed (Figure 5L). Hence, these findings provided critical evidence that a strong antitumor immune response has been established.

### 2.6 MDCa@RBC‐Alipo Nanobiologics Potentiates Checkpoint Inhibition

**Figure 6:**
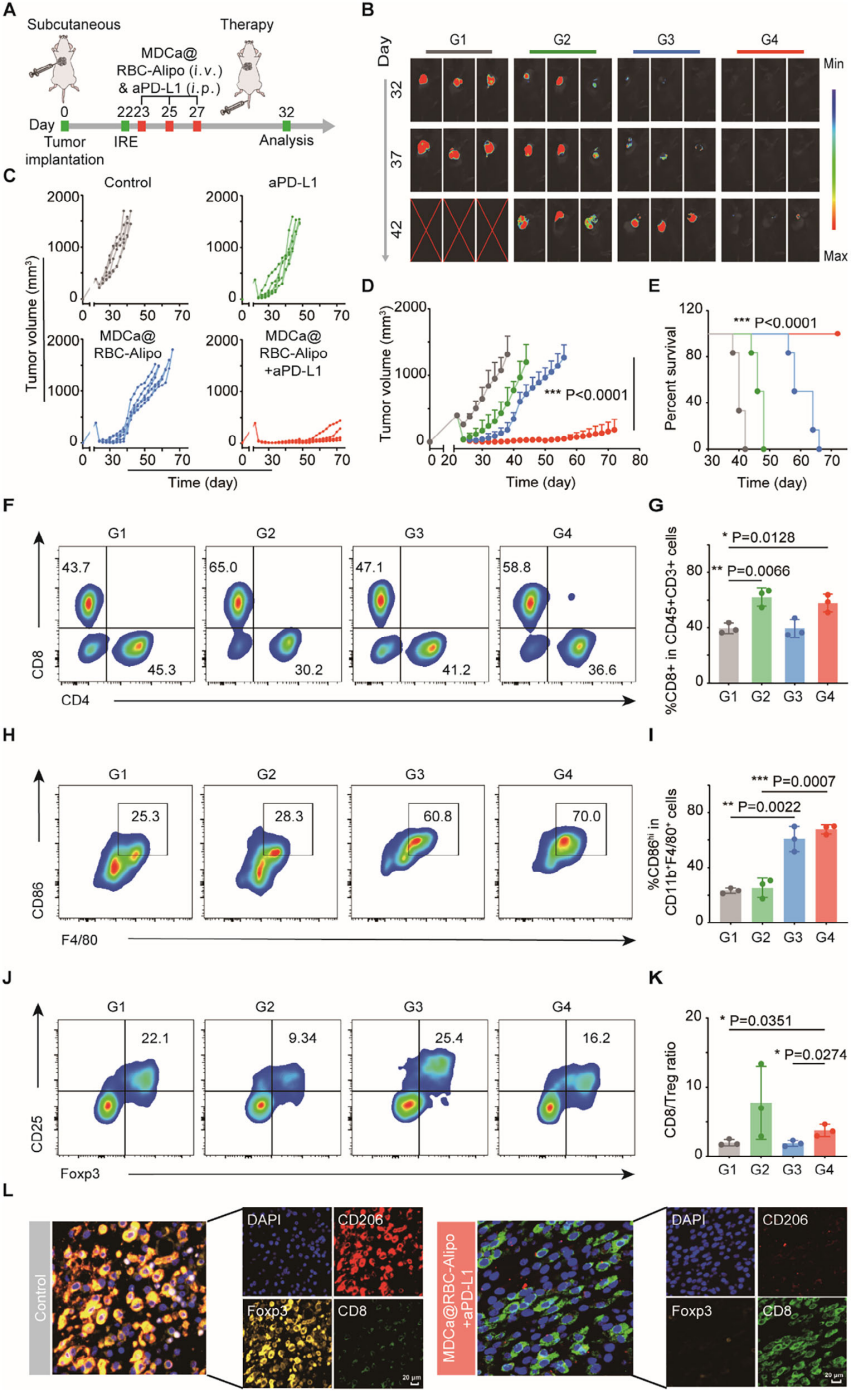
MDCa@RBC‐Alipo‐induced trained immunity synergizes with aPD‐L1 to trigger robust antitumor immune responses. A) Schematic illustration of the MDCa@RBC‐Alipo combined with anti‐PD‐L1 therapy in a subcutaneous tumor mouse model receiving incomplete irreversible electroporation (IRE) therapy. B) Representative bioluminescence images of Luc^+^Pan02 tumor after various treatments as indicated. C–E) Individual tumor growth kinetics (C), average tumor growth curves (D), and Kaplan–Meier survival curves (E) of Pan02 tumor‐bearing mice after varied therapeutic combinations (*n* = 5 in (C–E). F–K) Representative flow cytometric analysis and relative quantification of TAM‐M1 (CD86^hi^F4/80^+^CD11b^+^CD45^+^), CD8^+^ T cells (CD8^+^CD3^+^CD45^+^) and Treg (Foxp3^+^CD25^+^CD4^+^) in tumors. L) Polychromatic immunofluorescent staining images of tumors showing DAPI (blue), CD206^+^ (red), FoxP3^+^ (orange), and CD8^+^ (green) cells infiltration for Control and MDCa@RBC‐Alipo+aPD‐L1 groups. G1, Control; G2, aPD‐L1; G3, MDCa@RBC‐Alipo; G4, MDCa@RBC‐Alipo+aPD‐L1. Statistical difference was calculated using a two‐tailed unpaired student's *t‐*test. Data were expressed as means ± SD. *P < 0.05, **P < 0.01, and ***P < 0.001.

To evaluate the potential of utilizing trained immunity‐inducing nanobiologics as a combinatorial approach with checkpoint inhibition, various treatment regimens were assessed in C57BL/6 mice bearing the Pan02 PCa model. Twenty‐two days after tumor cell injections, mice were treated with IRE and then randomized and allocated one of four treatment groups (i.e., PBS, aPD‐L1, MDCa@RBC‐Alipo, MDCa@RBC‐Alipo+aPD‐L1). The aPD‐L1 was administered intraperitoneally at a dose of 3.75 mg kg^−1^ on days 23, 25, and 27 (**Figure** **6**A). The primary objective of these experiments was to compare the efficacy of aPD‐L1 immunotherapy administered as monotherapy versus its combination with our MDCa@RBC‐Alipo therapy, which induces trained immunity. Our findings showed that although aPD‐L1 monotherapy could somewhat suppress tumor growth, it is the case that all mice developed fatal tumors. Notably, MDCa@RBC‐Alipo nanobiologics combined with PD‐L1 blockade remarkably suppressed tumor growth (Figure 6B–D). Also, as shown by Figure 6E, the control group of mice exhibited a significantly shortened life span, with a median survival of only 40 days. The monotherapy, either aPD‐L1 or MDCa@RBC‐Alipo, exhibited a moderate enhancement in animal survival to 47 and 61 days. In stark contrast, the MDCa@RBC‐Alipo nanobiologics combined with PD‐L1 blockade exhibited a remarkable 72‐day survival rate, thereby highlighting the profound efficacy of combination therapy against tumor growth. Moreover, no abnormal weight loss of mice was observed in the MDCa@RBC‐Alipo plus aPD‐L1 group (Figure S18, Supporting Information).

After confirming that MDCa@RBC‐Alipo treatment effectively rebalances the immune cell populations within the tumor, thereby enhancing the susceptibility of the immune system to checkpoint blockade therapy, we employed FCM to further investigate alterations in leukocyte subpopulations resulting from the combined treatments. At day 32, tumor‐bearing mice were sacrificed, and single‐cell suspensions were obtained from the tumors for flow cytometry analysis. We observed that αPD‐L1 immunotherapy, as a monotherapy and in combination with MDCa@RBC‐Alipo treatment, significantly increased the CD8^+^ T cells in the tumors (Figure 6F,G). Concomitantly, TAMs‐M1 in tumors treated with MDCa@RBC‐Alipo+αPD‐L1 were substantially increased compared to those in the control ones (Figure 6H,I). Also, the antitumor immune balance indicators (e.g., M1/M2 and CD8/Treg) were obviously improved in the combination therapy group (Figure 6J,K; Figure S19, Supporting Information). These results were further confirmed by the mIHC analysis, which yielded consistent results (Figure 6L). Additionally, serum cytokine assays including TNF‐α, IL‐6, and IL‐1β showed that MDCa@RBC‐Alipo plus aPD‐L1 induced the highest levels of cytokine secretion in comparison to the PBS controls (Figure S20, Supporting Information). This demonstrates that innate immune system activation is crucial for the observed antitumor effects, but optimal therapeutic activity requires engagement with adaptive immune cells.

**Figure 7:**
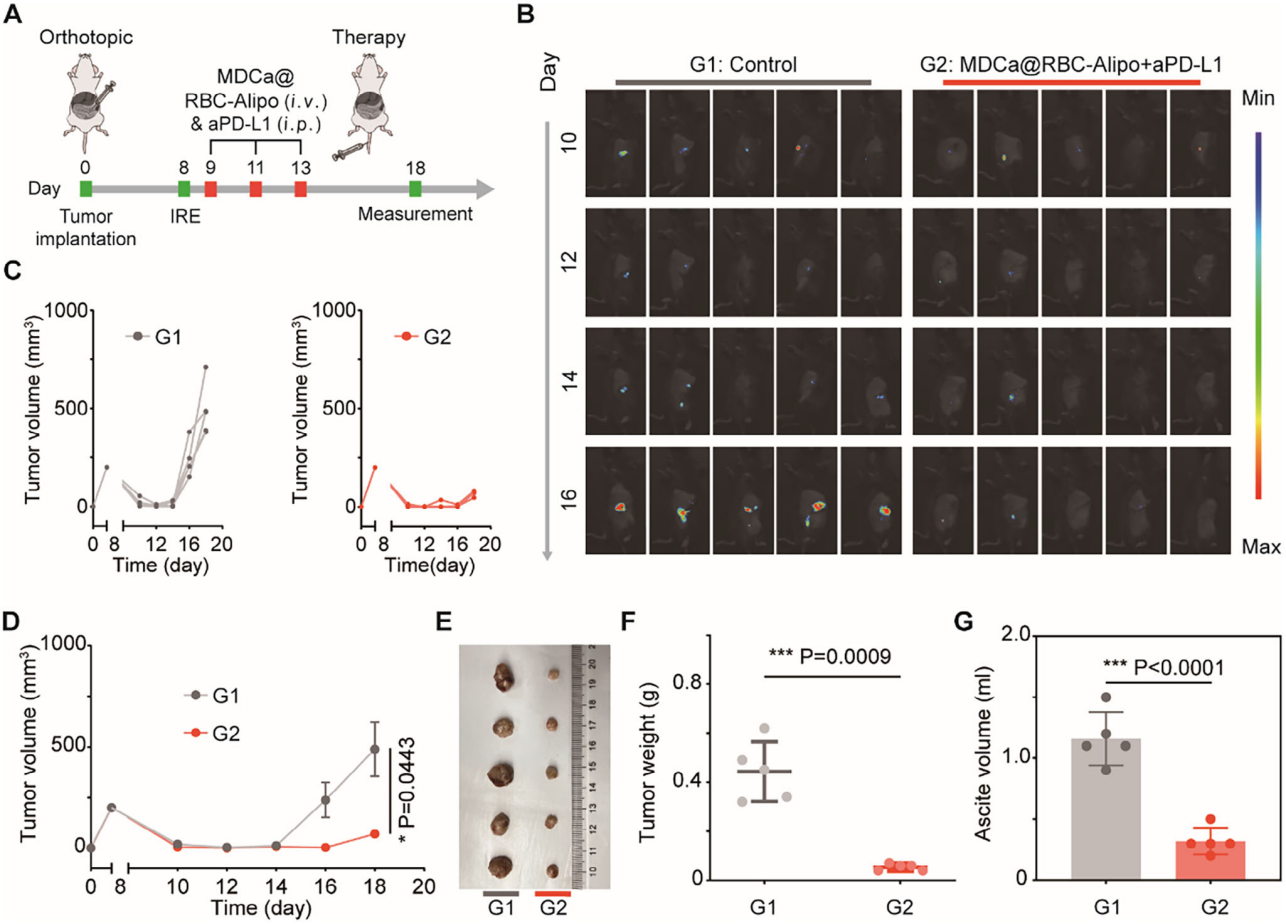
Combination therapy suppress postablative tumor progression in orthotopic models of PCa. A) Schematic illustration of the MDCa@RBC‐Alipo combined with anti‐PD‐L1 therapy in an orthotopic tumor mouse model receiving incomplete irreversible electroporation (IRE) therapy. B) Representative bioluminescence images of Luc+Pan02 tumor after various treatments as indicated. C,D) Individual tumor growth kinetics (C) and average tumor growth curves (D) of Pan02 orthotopic tumor‐bearing mice after varied therapy via bioluminescence images. E) Digital photo of tumors at the end of treatment. F) Average weights of tumors at the end of treatment (*n* = 5) G) The ascite volume of mice after various treatments. Statistical difference was calculated using a two‐tailed unpaired student's *t*‐test. Data were expressed as means ± SD. *P < 0.05, ***P < 0.001.

The aforementioned anti‐tumor evaluation was implemented on subcutaneously xenografted tumors. Furthermore, orthotopic Pan02 tumor models mimicking human PCa were employed to assess the anti‐tumor efficiency of MDCa@RBC‐Alipo plus PD‐L1 blockade. The schematic depicting the experimental procedures is shown in **Figure** **7**A. The tumor models were inoculated with Pan02 PCa cells that stably expressed luciferase, enabling sequential bioluminescent imaging of tumor progression (Figure 7B). With regard to antitumor response, the results on the orthotopic tumor model were approximately the same as those of the subcutaneously xenografted tumor model. The combination of MDCa@RBC‐Alipo and aPD‐L1 resulted in robust antitumor ability, due to the synergistic contributions from trained immunity‐inducing nanobiologics and PD‐L1 blockade‐mediated immunotherapy. Consequently, this distinctive treatment strategy resulted in the largest suppression rate and weight reduction of the orthotopic tumors (Figure 7C–F), and notably, it also significantly inhibits the development of hemorrhagic ascites (Figure 7G). The combined treatment led to a noticeable disparity in body weight among mice, which differed from the results obtained using a subcutaneous xenograft tumor model, primarily due to the suppression of hemorrhagic ascites development (Figure S21, Supporting Information). Additionally, the serum cytokine assays, including TNF‐α, IL‐6, and IL‐1β, revealed that MDCa@RBC‐Alipo combined with aPD‐L1 induced significantly higher levels of cytokine secretion compared to the PBS controls (Figure S22, Supporting Information). Also, the serum biochemistry assay and histology analysis of major organs obtained from mice 10 days after treatment revealed no evident toxic effects induced by this combined immunotherapeutic strategy (Figure S23, Supporting Information). Together, our results prove that the combined therapy strategy integrating MDCa@RBC‐Alipo‐induced trained immunity with anti‐PD‐L1 holds great potential in synergistic cancer immunotherapy.

## 3 Conclusion

As a crucial lymphoid organ responsible for producing immune responses to protect the body, the spleen harbors a large number of immune cells, cytokines, and other immune substances, which can be released into the peripheral blood to directly attack tumor cells and tissues. Consequently, targeting and stimulating robust immune responses in the spleen represents a viable approach for developing efficacious antitumor strategies. In this study, we initially delineate the reorganization of splenic immune cells, characterized by augmented frequencies of myeloid cells that concur with the progression of pancreatic carcinoma. Thus, we present a highly biocompatible nanobiologics that is formulated from natural molecular building blocks, namely, phospholipids, other fatty molecules, and apoA1, using a simple, fast, and reproducible formulation process. In mouse studies, we found that the nanobiologics rapidly accumulate in the spleen and display a strong affinity for myeloid cells. We demonstrate the ability to engage these splenic myeloid cells by epigenetic and metabolic rewiring, thereby facilitating efficient modulation of the peripheral trained innate immune response, as evidenced in both in vitro and in vivo experiments. Importantly, we exhibit that targeting peripheral trained immunity with nanobiologics can subsequently overcome the immunosuppressive TME in residual PCa post‐IRE treatment and potentiate the therapeutic effects of checkpoint inhibition. The induction of trained immunity in the adjuvant setting has been demonstrated to elicit a reduction in tumor size, suppression of hemorrhagic ascites development, and an extension of overall survival. Our carefully designed nanobiologics comprise erythrocyte membranes and surface‐functionalized apoA1‐based liposomes, which are inherently biocompatible and exhibit high in vivo tolerability. The monotherapy with MDCa@RBC‐Alipo or the combined treatment with aPD‐L1 did not affect liver function, renal function, and body weight. Therefore, the designed nanobiologics and the efficient combined therapeutic strategy are highly promising toward further clinical translation.

This study has several limitations. While orthotopic models better mimic tumor microenvironments than subcutaneous implants, they still lack key human stromal components. We should establish patient‐derived xenograft models to better predict clinical responses. While current good manufacturing practice‐compliant methodologies for erythrocyte membrane derivation have been established, achieving pharmaceutical‐grade batch consistency in nanoparticle drug loading remains a critical hurdle for scalable production.

In conclusion, our data presented here offer strong evidence for the complex reactions triggered in the spleen after tumor‐bearing. This can provide a new paradigm for promoting clinical translational therapy based on spleen immune regulation and is expected to accelerate the development and clinical application of the next generation of tumor immunotherapy. Our findings also highlight that the induction of peripheral trained immunity in the spleen could play a consequential role in reprogramming the suppressive TME of PCa, which could result in extending the life of patients diagnosed with this deadly malignancy.

## 4 Experimental Section

### Materials

All chemicals in this study were was purchased from Sigma‐Aldrich unless other instructions. The phospholipid 1,2‐dihexadecanoyl‐snglycero‐3‐phosphocholine (DPPC), cholesterol, 1,2‐distearoyl‐sn‐glycero‐3phosphoethanolamine‐N‐[amino (polyethylene glycol)2000] (DSPE‐PEG‐2000) and DSPE‐PEG‐Cy5.5 were obtained from Ruixi Biotechnology Co., Ltd (Xi'an, China). MDP was provided by Apeptide (Shanghai, China). The apoA1 was supplied by Beyotime Biotechnology (Shanghai, China). Anti‐PD‐L1 was purchased from Bioxcell (B7‐H1, Catalog No. BE0101).

### Cell Lines and Animals

The Pan02 cell line and J774A.1 cell line were originally obtained from the American Type Culture Collection (ATCC). Cells were authenticated via STR profiling and maintained in RPMI‐1640 or DMEM medium supplemented with 10% FBS at 37 °C under 5% CO₂ for use in this experiment. Female and male C57BL/6 mice aged six to eight weeks were utilized in all experiments and were procured from Shanghai Yuanchuang Biotechnology Co., Ltd. Mice were maintained on a 12‐h light/dark cycle with controlled humidity (≈55%). All animals were housed in a barrier facility, and only healthy mice were used for experiments. All procedures were approved by the Institutional Animal Care and Use Committee of the Laboratory Animal Center of Shanghai Tenth People's Hospital and complied with ARRIVE guidelines.

### Tumor‐Bearing Mouse Model Construction and Single‐Cell Sequencing

The tumor‐bearing mouse model was established by subcutaneously injecting a density of 1 × 10^6^ Pan02 cells into female C57BL/6 mice (6–8 weeks) on the left flank. Mice were monitored daily for tumor growth and euthanized when the tumor reached 100, 200, 400, 600, 800, and 1000 mm^3^. Following euthanization, the spleen of mice was harvested to observe their length and weight. Total RNA was extracted from the spleen tissue of tumor‐bearing mice with a volume of 400 mm^3^, as well as from time‐matched untreated mice, for subsequent sequencing analysis. After a sequence of complex extraction processes, the total RNA was quantified and identified by BD Rhapsody WTA Analysis Pipeline(version 1.8). Graph‐based clustering was performed to cluster cells according to their gene expression profiles using the pp.neighbors function in Scanpy. The clustering of the gene of spleen from untreated and tumor‐bearing groups was identified, and the data was shown as mean values in the different illustrations. Besides, the R package g: Profiler to perform functional enrichment analysis of marker genes was used. The marker genes for each cluster were mapped to Gene Ontology pathways, and the hypergeometric distribution was used to check whether these biological processes were over‐represented.

### Synthesis of MDCa@RBC‐Alipo

DPPC, DSPE‐PEG‐2000, cholesterol, and apoA1 with a fixed weight ratio of 3:1:1:1.5 were added into the trichloromethane solution. According to the reverse evaporation method, chloroform was evaporated under 100 bar and 100 rpm at 60 °C for 1 h to form a thin lipid film. Then, 5 ml of PBS (0.01 M, pH = 7.6) was added, and the opaque solution was rotated at 100 rpm at 37 °C in an oil bath for 1 h. The alipo vesicles were obtained via extruded 20 times at 400 nm and 20 times at 200 nm. To incorporate DiOC18 dyes in the nanobiologics, the desired dye (0.5 mg) was dissolved in the chloroform solution utilized for lipid film preparation.

The membrane vesicles of RBC were harvested by the hypotonic burst method. The pure red blood cells were obtained by washing and centrifuging the whole blood of mice three times. Subsequently, the precipitated red blood cells were resuspended in 3× volume of pure red blood and pre‐cooled with 0.25× PBS, followed by incubation at 4 °C for 25 min to induce plasmatorrhexis. The supernatant was clarified through three rounds of repetitive centrifugation at 12 000 rpm and 4 °C for 10 min each. The precipitation was resuspended using precooled PBS, and the erythrocyte membrane vesicles were harvested by extruding them 20 times through mini‐extruders equipped with a 400 and 200‐nm membrane. The erythrocyte membrane vesicles were mixed with Alipo vesicles in a ratio of 1:1, using a tip sonicator at 4 °C at 100 W for 10 min. The RBC‐Alipo fusion membrane was harvested by mini‐extruders using a 400 and 200 nm membrane for 20 times.

To obtain MDCa, 1 ml HEPES saline buffer (50 mm, pH 7.1) containing 100 µg MDP, 10 mg PEG‐b‐P(Glu) block copolymers (synthesized as previously described48), and 10 mm Na_2_CO_3_ was mixed with 1 ml of Tris‐HCl buffer (1 mm, pH 7.6) containing 100 mm CaCl_2_. The mixture was stirred at 4 °C for 12 h. Excessions, copolymers, and medicine were cleared by centrifugation at 14 800 rpm for 10 min. Ultimately, ultrasonic extrusion was used for the fusion of MDCa and RBC‐Alipo membrane. After putting the mixture into a tip sonicator at 4 °C at 100 W for 10 min, the MDCa@RBC‐Alipo was obtained by extrusion at 400 nm and at 200 nm for 20 times.

### Characterizations

Transmission electron microscopy (TEM) was conducted on a JEM‐2100F instrument. MDCa@RBC‐Alipo samples were deposited on carbon‐coated grids, negatively stained with 2% phosphotungstic acid (pH 6.8) or 1% uranyl acetate, and vacuum‐dried. Imaging at 200 kV using HAADF detected structural features. Dynamic light scattering (DLS, Malvern Nano ZS90) measured hydrodynamic diameter (Z‐average) and zeta potential in PBS (50–200 µg mL^−1^, 0.22 µm‐filtered) at 25 °C with triplicate measurements.

Encapsulation efficiency (EE) of MDP was quantified via colorimetric detection of N‐acetylglucosamine (GlcNAc) using the Elson–Morgan assay. A standard curve (0–100 µm GlcNAc) was established by reacting samples with 200 µL Elson–Morgan reagent (4% p‐dimethylaminobenzaldehyde in Hc), followed by 10 min heating at 100 °C and absorbance measurement at 540 nm (*R* ^2^ > 0.99). MDCa@RBC‐Alipo was lysed (200 W sonication, 5 min) and dissolved with HCl (1000 g, 10 min). Free MDP in the supernatant was separated using 100 kDa ultrafiltration (4000 g, 20 min) to further detect the MDP content in nanoparticles. EE% was calculated as M2/M1 × 100%, with triplicate samples and spiked recovery controls (M2: Drug content in nanoparticles; M1: Dosage).

### Degradation and Drug Release Studies

The release of MDP in vitro was studied at 37 °C by mixing MDCa@RBC‐Alipo with PBS (pH = 5.0), PBS (pH = 6.5), or PBS (pH = 7.4) under shaking. At pre‐set time points, released MDP was measured by colorimetric assay.

### Myeloid Cells Isolation

The spleen‐derived myeloid cells were acquired according to the protocols of Junyu Lu and Yiping Wang.^[^ ^43^ ^]^ The C57BL/6 mouse splenocytes were aseptically harvested and mechanically dissociated using sterile syringes. Subsequently, the resulting cell suspension was passed through a 40 mm nylon mesh filter and incubated at 37 °C for 40 min. Following this, the culture supernatant containing floating cells (T cells, dendritic cells, and natural killer cells) was discarded. The adherent spleen‐derived macrophages were washed three times in the RPMI‐1640 medium and cultured with the standard medium.

### RNA Sequencing

Following administration of PBS or MDCa@RBC‐Alipo, spleens were harvested after seven days and processed into a single‐cell suspension. Single‐cell RNA sequencing libraries were prepared using the BD Rhapsody whole transcriptome analysis (WTA) Pipeline (v1.8) without targeted panel selection, following microdroplet‐based single‐cell capture and cDNA amplification protocols. Sequencing was performed on an Illumina NovaSeq 6000 platform (specify read length, e.g., 150 bp paired‐end). Raw FASTQ files were processed through the BD Rhapsody pipeline with alignment to species‐specific reference genomes (mouse: mm10) and transcriptome annotations (mouse: GENCODE vM23/Ensembl 98), generating a UMI count matrix.

Low‐quality cells were filtered using thresholds of ≤100 000 total UMIs, ≤8000 detected genes per cell, and >10% mitochondrial gene content. Post‐QC, 14129 cells were retained. Data normalization was performed in Scanpy (v1.8) by scaling counts to 10 000 transcripts per cell (scanpy.pp.normalize_total) followed by log1p transformation. The top 2000 highly variable genes were selected using Macosko's dispersion method (scanpy.pp.highly_variable_genes).

Principal components (top 50 PCs) derived from PCA (scanpy.tl.pca) were used to construct a nearest‐neighbor graph (scanpy.pp. neighbors, n_neighbors = 15) and cluster cells via the Leiden algorithm (scanpy.tl. leiden, resolution = 0.6). Dimensionality reduction was visualized using UMAP (scanpy.tl. umap, min_dist = 0.5). Cluster‐specific marker genes were identified with Seurat's Wilcoxon test (FindAllMarkers, adj. *P* < 0.05, |log2FC| > 2). Functional enrichment analysis of markers was conducted via g: Profiler (FDR < 0.05) against GO, KEGG, Reactome, and WikiPathways databases to further elucidate the biological characteristics of the genes under the MDP versus PBS conditions and their disparities.

### In Vitro Phagocytosis Assay

J774A.1 cells (4 × 10^5^) were cultured in the serum‐free medium by adding various Cy5.5‐labeled nanoparticles. After incubation for 24 h at 37 °C, phagocytosis was investigated by confocal microscopy (Zeiss LSM 710).

### Confocal Imaging

For the cell uptake experiments, the J744A.1 cell nucleus was labeled with Hoechst, and the liposome was labeled with Cy5.5. After co‐incubating with nanoparticles, each dish was washed three times. The results were observed by inverted fluorescence microscopy at 480 and 530 nm.

### Biodistribution

The nanoparticles were labeled with Cy5.5 to prepare Cy5.5‐ MDCa@Lipo, Cy5.5‐MDCa@RBC‐Lipo, and Cy5.5‐MDCa@RBC‐Alipo. An IVIS (In Vivo Imaging System) spectral imaging system was used at 2, 4, 6, 8, 10, 12, and 24 h after intravenous injection of 100 µL nanoparticles (dose of MDP = 1.5 mg kg^−1^). The exposure time of bioluminescence imaging was 15 s. The average radiance of fluorescence intensity (s^−1^ cm^−2^ sr^−1^) was quantitatively analyzed via living image software. To analyze the distribution in isolated organs of nanoparticles, DiO‐MDCa@RBC‐Alipo was injected, and 6 hours later major organs were harvested and assessed by FCM.

### Cellular Specificity

For cellular specificity, C57BL/6 mice were intravenously injected with DiO‐MDCa@RBC‐Alipo for 24 h. Subsequently, mice were sacrificed, and single‐cell suspensions were harvested from the spleen. Cell suspensions were incubated with CD11b‐PE‐cy7 (BD, Catalog: 250112–82), and subsequently washed and resuspended in FACS‐buffer. All data were acquired on a flow cytometry instrument (BD, Fortessa X20). DiO‐MDCa@RBC‐Alipo was detected in the FITC channel. Additionally, the spleen sections were subjected to staining using a multiplex fluorescence immunohistochemical staining kit following the provided instructions in order to visualize the co‐localization of CD11b and DiO‐MDCa@RBC‐Alipo.

### In Vivo Restimulation Assay

After euthanasia, mouse spleens were harvested and placed in 5 mL of Hank's Balanced Salt Solution (HBSS), followed by gentle dissection into smaller pieces. They were suspended in a 5 mL HBSS solution containing type IV collagenase (100 U mL^−1^) and 1% FBS DNase (20 µg mL^−1^) at 37 °C for 20–30 min. The tissue was subsequently passed through a cellular screen to obtain a cell suspension. The cells were centrifuged at 400–600 × *g* for 5 min and the supernatants were discarded. Then the cells were suspended with 5 mL of pre‐cooled 1× RBC lysate for 5 min. After washing, suspending, and centrifuging, the macrophages were harvested with a concentration of 2–3 × 10^6^ cells mL^−1^.

### In Vivo Tumor Models and Treatment

To evaluate the therapeutic effects of MDCa@RBC‐Alipo, C57BL/6 mice (6–8 weeks old) were inoculated with Luc^+^ Pan02 cells (1 × 10^6^) on the left flank. Upon reaching a subcutaneous tumor volume of ≈400 mm^3^ (on day 22 after implantation), the mice bearing tumors were randomly assigned to one of four groups for treatment: control group, RBC‐Alipo group, MDCa group, and MDCa@RBC‐Alipo group. An incompletely ablated tumor model was created by using an IRE therapy device (Nanosecond pulsed tumor ablation system, Hangzhou Ruidi Biotechnology Ltd., Hangzhou, China) The electroporation procedure was conducted using a two‐needle array electrode featuring an 8 mm gap. The electric pulses were generated using the following parameters: a voltage of 1200 V, a pulse duration of 100 µs, a pulse repetition frequency of 1 Hz, and a total number of 99 repetitive pulses. After the ablation, the nanoparticles were injected on days 23, 25, and 27 via the tail vein. After ablation, the body weight and tumor size of mice were carefully measured by electronic balance and digital caliper every other day. An in vivo fluorescence imaging system was used to evaluate the tumor burden. After injecting *D*‐Luciferin (20 mg mL^−1^, at a dose of 5 µL g^−1^) into mice and waiting for eight minutes, the image was captured within a 30‐second exposure period. The tumors were further collected for flow cytometry analysis and polychromatic immunofluorescent staining.

To evaluate the efficacy of combination therapy involving anti‐PD‐L1, the aforementioned procedures were followed to establish a subcutaneous tumor‐bearing mouse model. Tumor‐bearing Mice were randomly divided into four groups, including a control group, an anti‐PD‐L1 group, an MDCa@RBC‐Alipo group, and an MDCa@RBC‐Alipo + aPD‐L1 group. After the ablation, the nanoparticles were injected via the tail vein, and anti‐PD‐L1 (3.75 mg kg^−1^) was intraperitoneally administered on days 23, 25, and 27. After ablation, the body weight and tumor size of mice were carefully measured by electronic balance and digital caliper every other day. An in vivo fluorescence imaging system was used to evaluate the tumor burden.

To evaluate the efficacy of combination therapy in the orthotopic pancreatic tumor‐bearing mouse model, Luc^+^ Panc02 cells (cultured in DMEM/10% FBS) were suspended in PBS/Matrigel (1:1) at 1 × 10⁶ cells/50 µL. C57BL/6 mice (6–8 weeks old) were anesthetized with isoflurane, and a laparotomy exposed the pancreas. Cells were injected into the pancreatic parenchyma. Incisions were sutured with absorbable material. Tumor growth was tracked every other day by bioluminescence imaging. The ablation treatment began when the volume of the orthotopic tumor approximately reached 200 mm^3^ on day 8. Mice were randomly divided into two groups including the PBS group and the MDCa@RBC‐Alipo + aPD‐L1 group, which received injections on days 9, 11, and 13. When the tumor volume exceeded 500 mm^3^ or cachexia signs appeared, mice were euthanized.

### Western Blot Procedures

The equivalent protein (using the Enhanced BCA Protein Assay Kit, Beyotime, P0010) was mixed with SDS Page loading buffer (Beyotime, P0015L) and incubated at 100 °C for 10 min. After gel electrophoresis, protein transfer and blocked, the protein was incubated overnight with 1:1000 diluted Histone H3 mouse monoclonal antibody(Beyotime, AF0009), Acetyl‐Histone H3 (Lys27) rabbit monoclonal antibody (Beyotime, AG3899), Tri‐Methyl‐Histone H3 (Lys4) mouse monoclonal antibody(Beyotime, AG3833) and GAPDH mouse monoclonal antibody(Beyotime, AF0006) at 4 °C. Then the membrane was washed 3 times and incubated with 1:5000 diluted anti‐mouse lgG‐HRP secondary antibody(Beyotime, A0216) or anti‐rabbit lgG‐HRP secondary antibody (Beyotime, A0208) for 1 h at room temperature. Detection was captured by an automatic chemiluminescence image analysis system (Tanon 4600).

### Cytokine Detection

The concentration of bioactive TNF‐α, IL‐6, IL‐1β, and IL‐10 in cell culture supernatant and serum was tested by enzyme‐linked immunosorbent assay (ELISA) kits according to the manufacturer's instructions. All the test kits were purchased from MultiSciences.

### Flow Cytometry Analysis

The dissociated tumor specimens and splenic tissues were enzymatically processed to obtain single‐cell suspensions. Following centrifugation, cells were initially treated with a CD16/32 blocking antibody (eBioscience, clone FRC‐4G8, Cat# MFCR00) through a 15‐minute incubation at 4 °C to prevent non‐specific antibody interactions. Subsequently, the prepared cell samples were subjected to staining procedures using optimally diluted fluorescently labeled antibodies under light‐protected conditions. The antibodies involved in the experiment include: CD45‐eF506 (eBioscience, Catalog: 69‐0451‐82), CD11b‐PE‐cy7 (eBioscience, Catalog: 250112–82), F4/80‐APC (eBioscience, Catalog: 17‐4801‐82), CD86‐FITC (eBioscience, Catalog: 11‐0862‐82), CD206‐PE (eBioscience, Catalog: 12‐2061‐82), CD3‐PE‐Cy7 (eBioscience, Catalog: 25‐0031‐82), CD4‐FITC (eBioscience, Catalog: 11‐0041‐82), CD8‐PerCP‐Cy5.5 (eBioscience, Catalog: 45‐0081‐82), CD25‐APC (eBioscience, Catalog: 17‐0251‐82), Foxp3‐PE (eBioscience, Catalog: 12‐5773‐82). The experimental procedures employed specialized reagents including erythrocyte lysis buffer (Thermo, Cat#00‐4300‐54) and fixation/permeabilization solutions from the intracellular staining kit (Thermo, Cat#888824‐00). Foxp3 nuclear staining was performed using the transcription factor detection kit (Thermo, Cat#00‐552300). All immunostaining antibodies were titrated to an optimal working concentration of 0.2 µg per experimental sample. Following staining protocols, cell suspensions were filtered through a 70‐µm mesh before multiparametric analysis using a BD Fortessa X20 flow cytometer. Acquired data were processed with FlowJo analytical software (version 10.6.2, TreeStar Inc.) for population quantification and visualization.

### Polychromatic Immunofluorescent Staining

The spleen and tumor sections were stained by the instructions of a multiplex fluorescence immunohistochemical staining kit (Absin, Catalog: abs50028). The antibodies referred to the experiment include CD206 (CST, Catalog: 24 595), CD8 (CST, Catalog: 98 941), Foxp3 (abcam, Catalog: ab215206), CD3 (CST, Catalog: 26 582), and TNF‐α (CST, Catalog, 57 485). The nuclei were stained with DAPI first, and all sections were analyzed by a fluorescent scanning camera (KFBIO, KF‐TB‐400).

### Statistical Analyses

The quantitative results were expressed as mean ± SD. All statistical differences were performed by GraphPad Prism software to use Student's *t‐*test for normal test. The survival curve was performed by the Log‐rank test. The statistical significance was set as ns, not significant, *P < 0.05, **P < 0.01, ***P < 0.001.

## Conflict of Interest

The authors declare no conflict of interest.

## Supporting information



Supporting Information

## Data Availability

The data that support the findings of this study are available on request from the corresponding author. The data are not publicly available due to privacy or ethical restrictions.
