# Supplementary material for: Targeting Splenic Myeloid Cells with Nanobiologics to Prevent Postablative Pancreatic Cancer Recurrence via Inducing Antitumor Peripheral Trained Immunity
Source: Adv Sci (Weinh). 2025 Apr 28;12(21):2413562. doi: 10.1002/advs.202413562 (PMC12140294; doi:10.1002/advs.202413562)
Supplement: Supplementary file 1 — Supporting Information [file ADVS-12-2413562-s001.docx]

Supplementary Materials for

Targeting splenic myeloid cells with nanobiologics to prevent postablative pancreatic cancer recurrence via inducing antitumor peripheral trained immunity

Shengbo Wu, Weichen Xu, Xuexia Shan, Liping Sun, Shuo Liu, Xixi Sun, Shaoyue Li, Xiaodong Hou, Xiaowan Bo, Chengzhong Peng, Bin Huang, Huixiong Xu, Wenwen Yue

Correspondence to: [hb2k@163.com](mailto:hb2k@163.com); [xu.huixiong@zs-hospital.sh.cn](mailto:xu.huixiong@zs-hospital.sh.cn); [yuewen0902@tongji.edu.cn](mailto:yuewen0902@tongji.edu.cn)


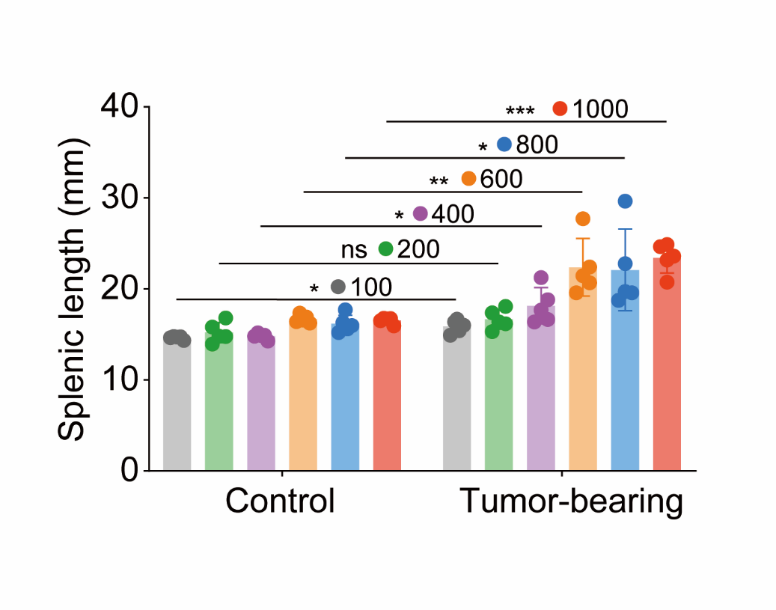


**Supplementary Figure 1**. Splenic length in mice bearing tumors of varying volumes (100-1000 mm^3^). Data were presented as means±SD (n=4-5). *P < 0.05, **P < 0.01 and ***P < 0.001.


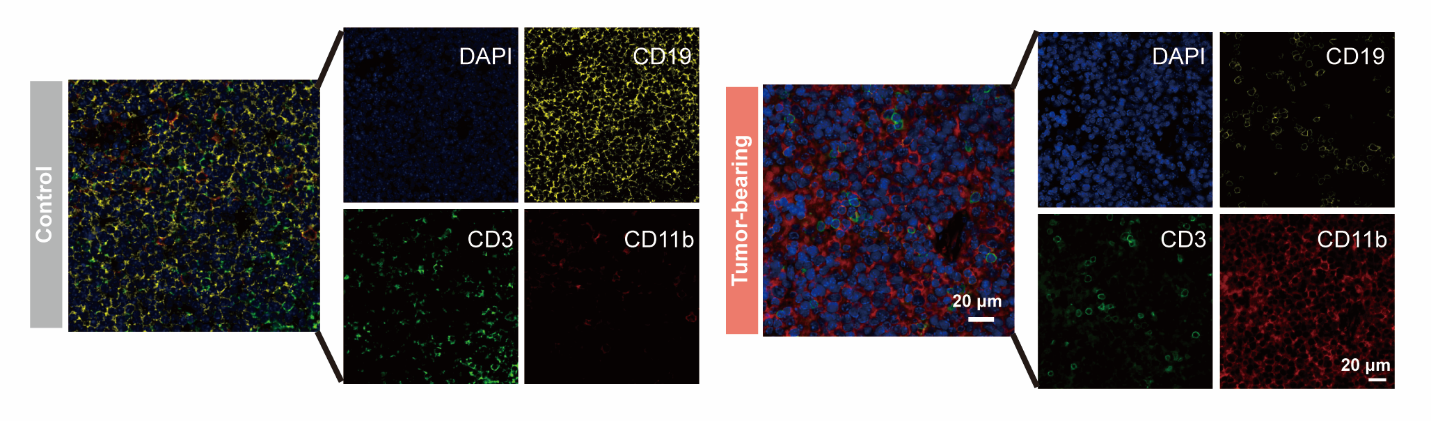


**Supplementary Figure 2.** Polychromatic immunofluorescent staining images of the spleen showing DAPI (blue), CD19^+^ (yellow), CD3^+^ (green), and CD11b^+^ (red) cell infiltration in both control and orthotopic pancreatic tumor-bearing mice.


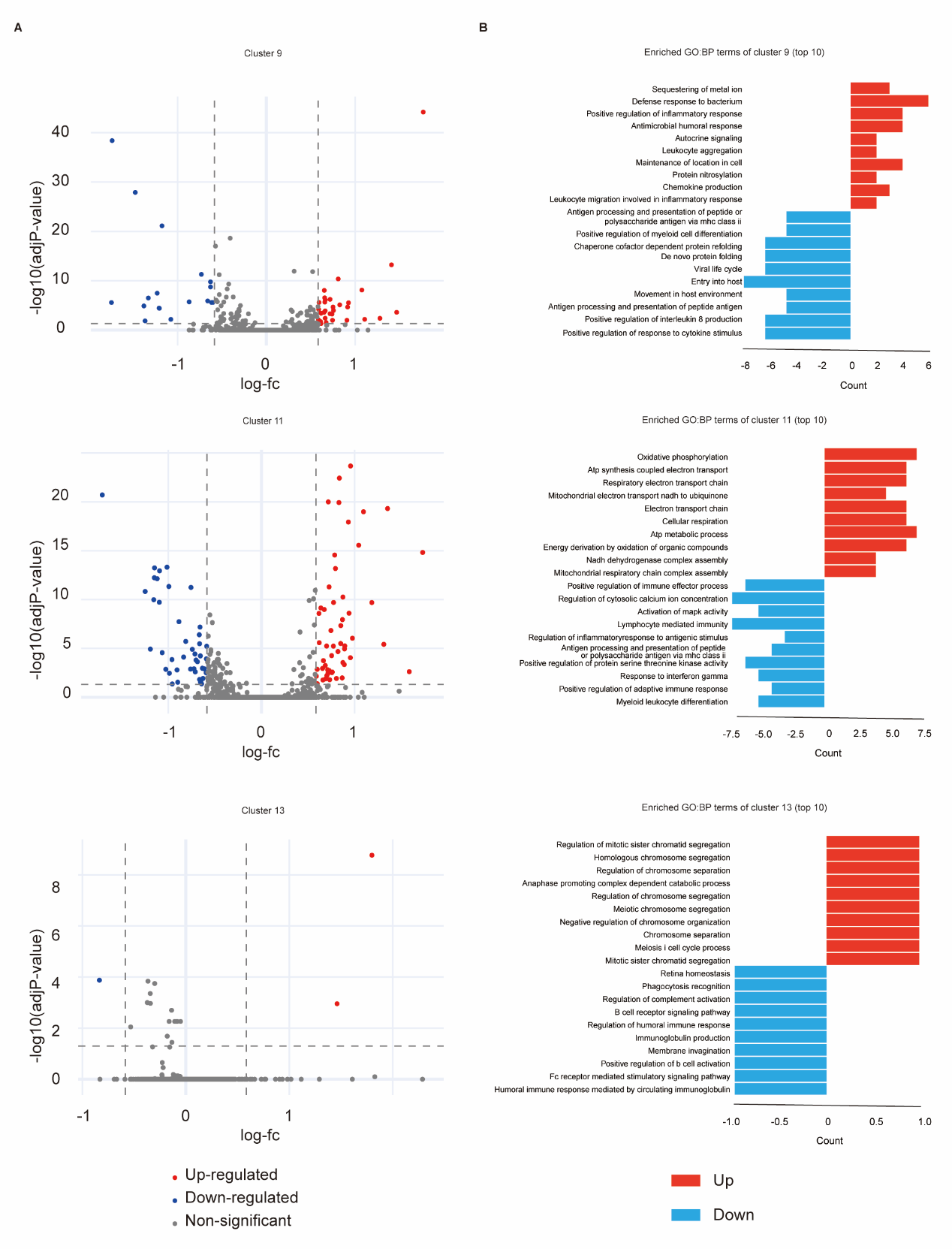


**Supplementary Figure 3**. **(A)** Volcano plots of splenic gene expression differences in clusters 9, 11 and 13 between untreated and tumor-bearing samples. Blue, red and grey colors indicate up- and down-regulated and non-significant genes, respectively. **(B)** Importantly and significantly different Gene Ontology (GO) pathway analysis of clusters 9, 11, and 13.


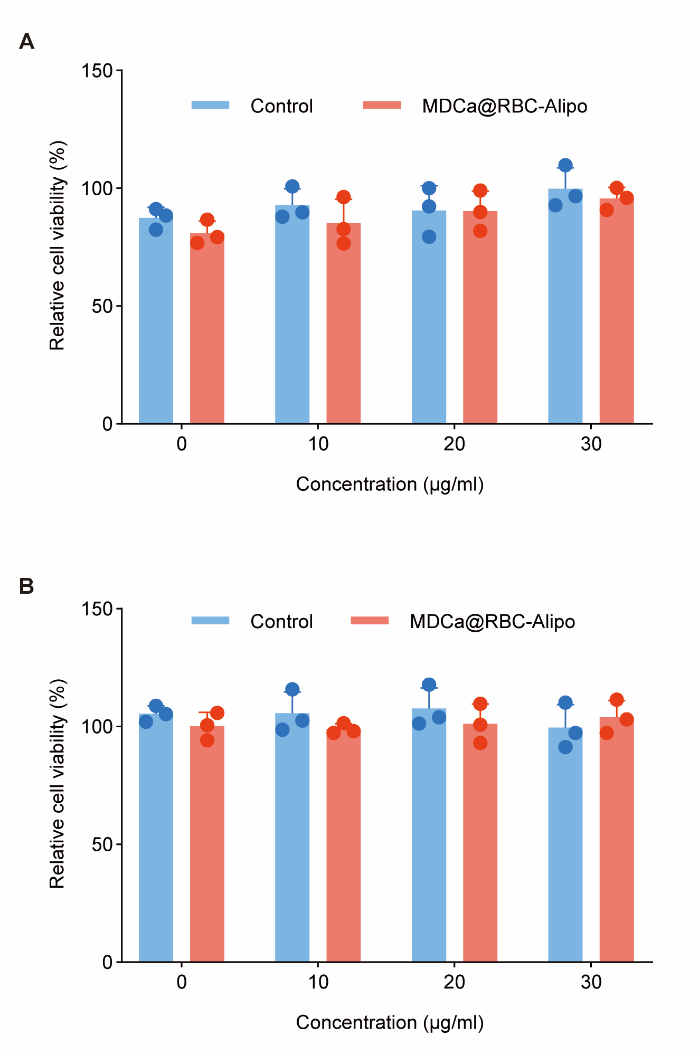


**Supplementary Figure 4. (A)** Cytotoxicity assay of J774A.1 cells preincubated with MDCa@RBC-Alipo at various concentrations of MDP. **(B)** Cytotoxicity assay of Pan02 cells preincubated with MDCa@RBC-Alipo at various concentrations of MDP. Data were presented as means±SD (n=3).


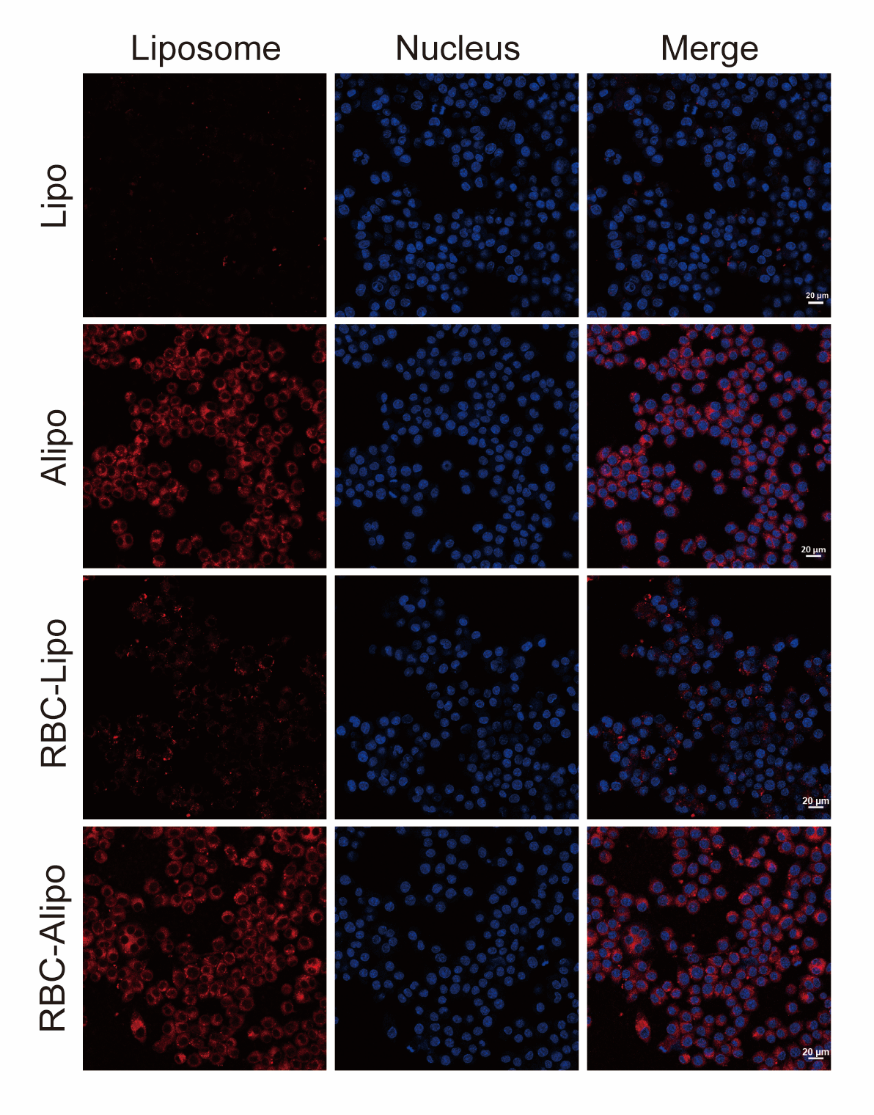


**Supplementary Figure 5**. Representative confocal laser scanning microscopy of endocytosis-dependent uptake of nanoparticles (Cy5.5-DSPE-PEG) in J774A.1 cells loaded with or without apoA1. Scale bars, 20 μm (n=3).


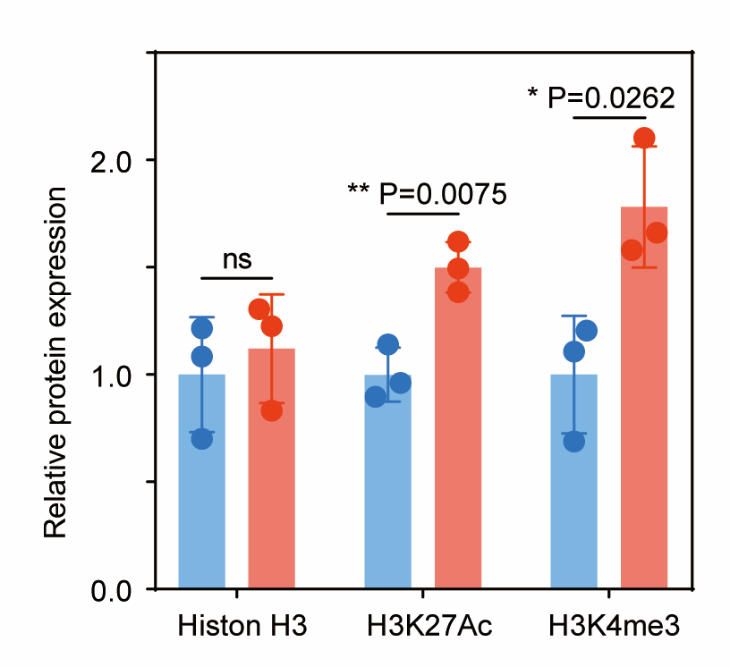


**Supplementary Figure 6**. Quantitative analysis of Histon H3, H3K27Ac and H3K4me3 western blot of J774A.1 cells preincubated with PBS or MDCa@RBC-Alipo for 24 h. Data were expressed as means ± SD (n=3) . ns, not significant, *P < 0.05 and **P < 0.01.


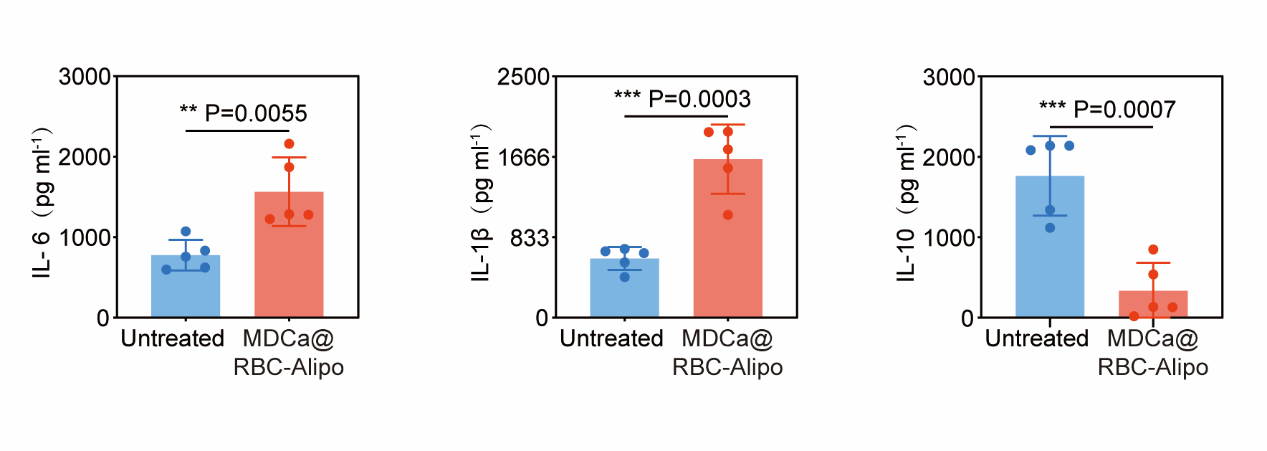


**Supplementary Figure 7**. Seven days after preincubated with PBS or MDCa@RBC-Alipo, J774A.1 cells were restimulated with LPS for 24 h. The medium was subjected to the multiplex cytokine (i.e., IL-6, IL-1β, and IL-10) analysis using ELISA. Data were presented as means±SD (n=5). **P < 0.01 and ***P < 0.001.


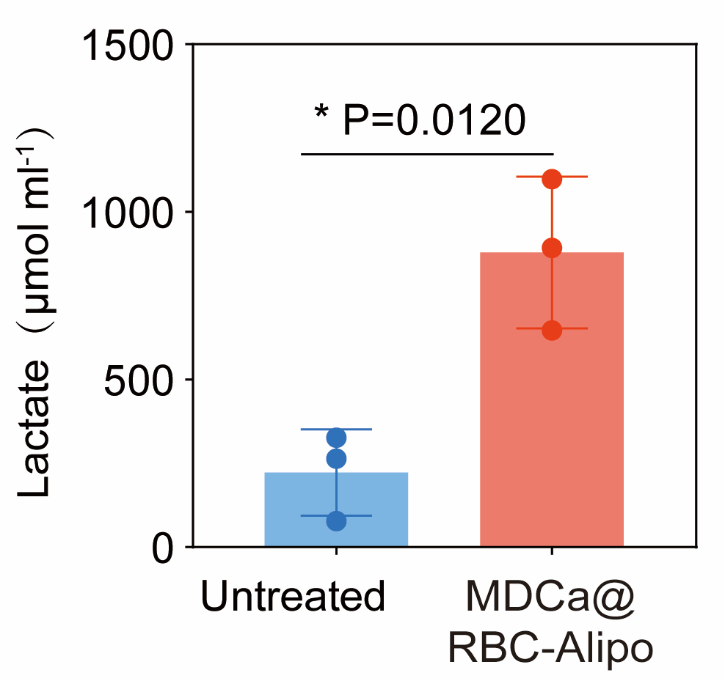


**Supplementary Figure 8**. ELISA was performed to measure the lactate levels in the cell supernatant of J774A.1 cells preincubated with either PBS or MDCa@RBC-Alipo for 24 hours. Data were presented as means±SD (n=3). *P < 0.05.


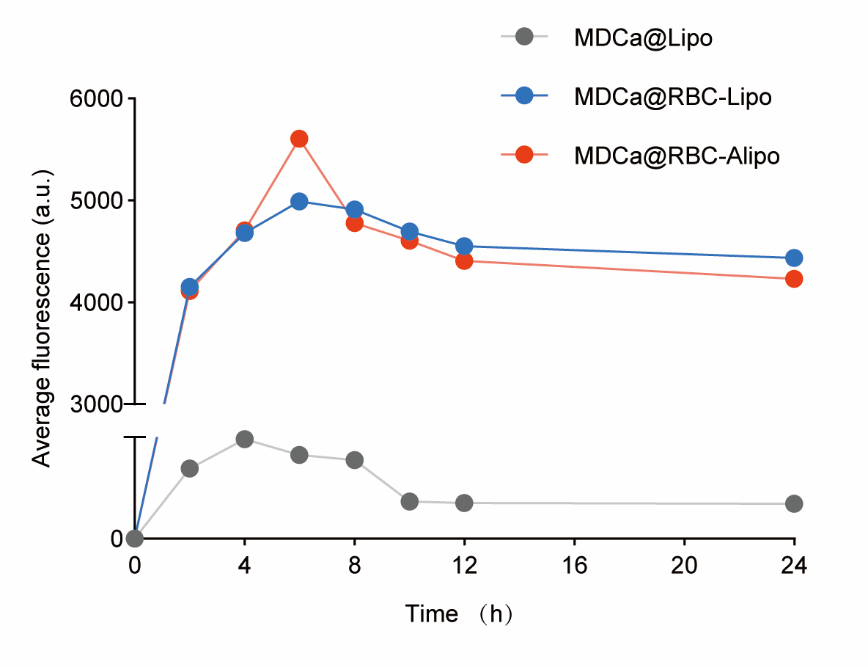


**Supplementary Figure 9**. The fluorescence intensity of the spleen was measured at various time points in vivo following intravenous administration of different types of nanobiologics (i.e., MDCa@Lipo, MDCa@RBC-Lipo, and MDCa@RBC-Alipo), as per the protocol.


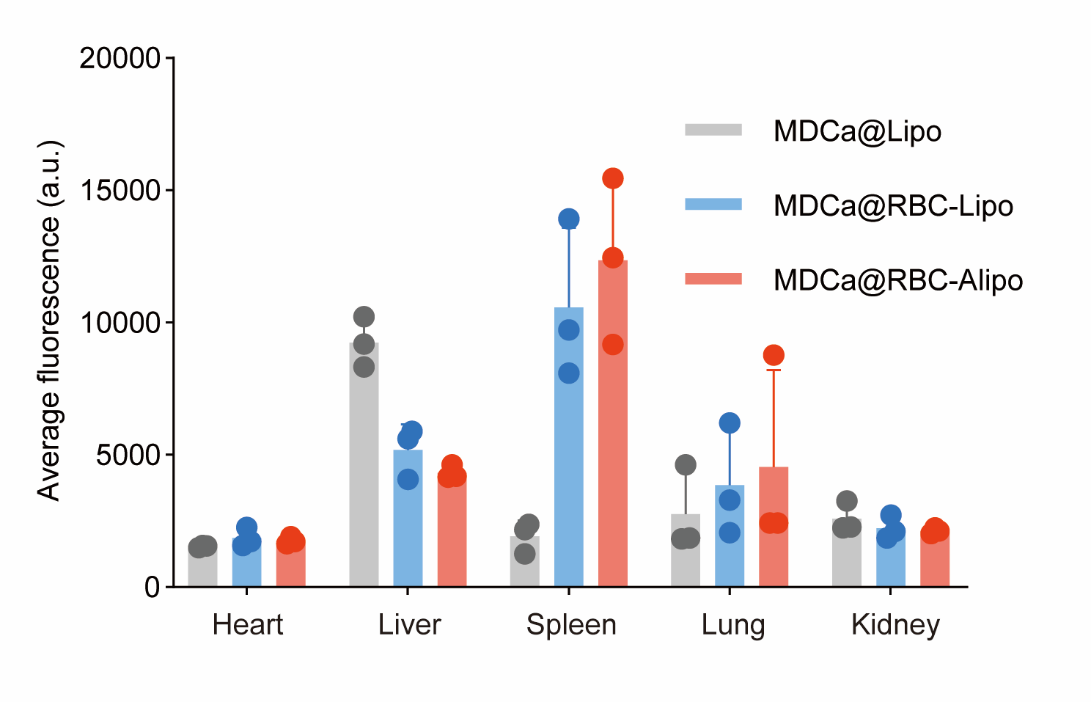


**Supplementary Figure 10**. The average fluorescence intensity of dissected organs was measured at 6 hours post-intravenous administration of various Cy5.5-labeled nanobiologics, including MDCa@Lipo, MDCa@RBC-Lipo, and MDCa@RBC-Alipo.


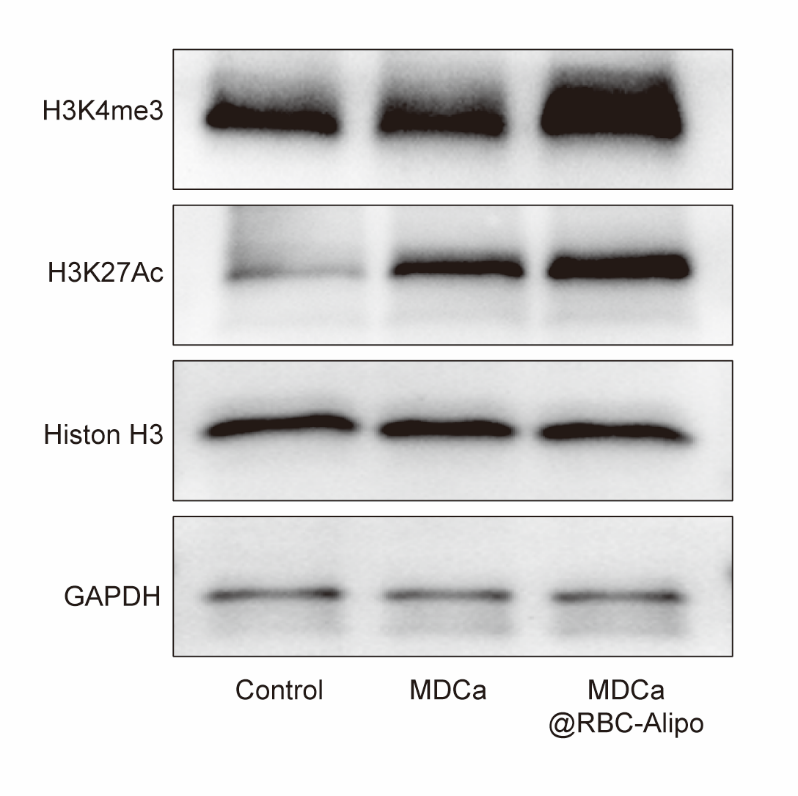


**Supplementary Figure 11**. The histones from splenic tissue of mice were isolated and subjected to western blot analysis after intravenous injection with PBS, MDCa, or MDCa@RBC-Alipo (n=3).


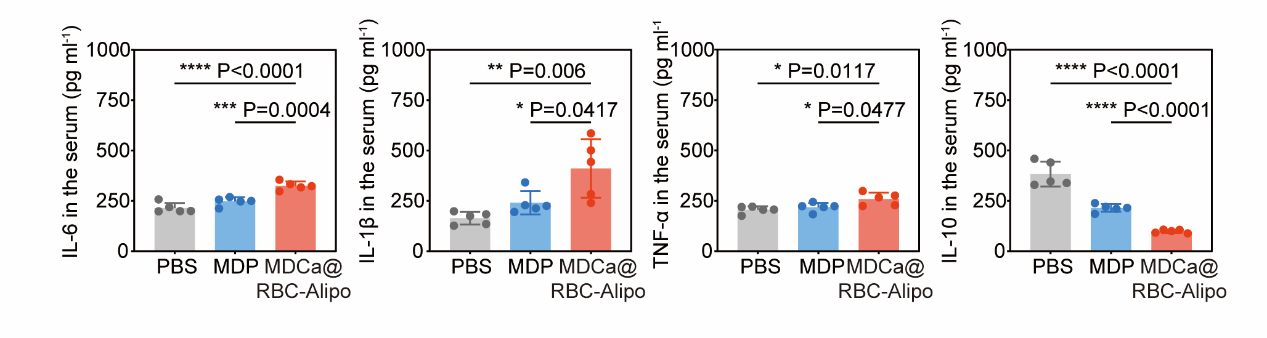


**Supplementary Figure 12**. Seven days after intravenous injection of PBS, MDCa, or MDCa@RBC-Alipo, myeloid cells in spleen were sorted and restimulated with LPS. IL-6, IL-1β, TNF-α and IL-10 in the supernatants were measured using ELISA. Data were presented as means±SD (n=5). *P < 0.05, **P < 0.01 and ***P < 0.001.


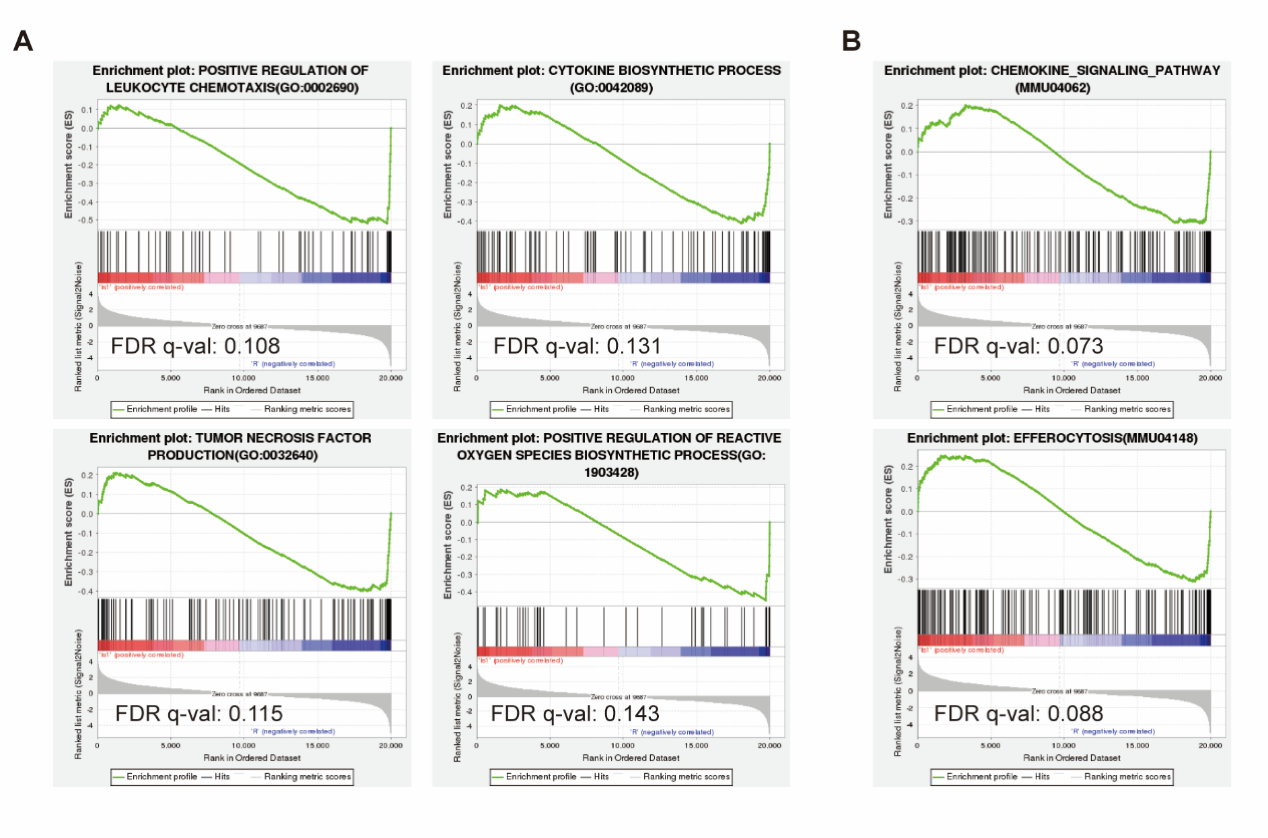


**Supplementary Figure 13**. **(A)** After a seven-day period following intravenous injection of MDCa@RBC-Alipo or PBS, splenic CD45^+^CD11b^+^ populations were sorted and subjected to RNA-Seq analysis. Enrichment plots generated by Gene Set Enrichment Analysis (GSEA) were utilized to investigate the GO terms associated with cytokine and TNF-α secretion, leukocyte chemotaxis, and reactive oxygen species biosynthetic processes in CD11b^+^ cells from the MDCa@RBC-Alipo-trained group compared to the PBS control. **(B)** GSEA was performed to analyze the KEGG pathways associated with efferocytosis and chemokine signaling in CD11b^+^ cells isolated from MDCa@RBC-Alipo-treated mice compared to those treated with PBS.


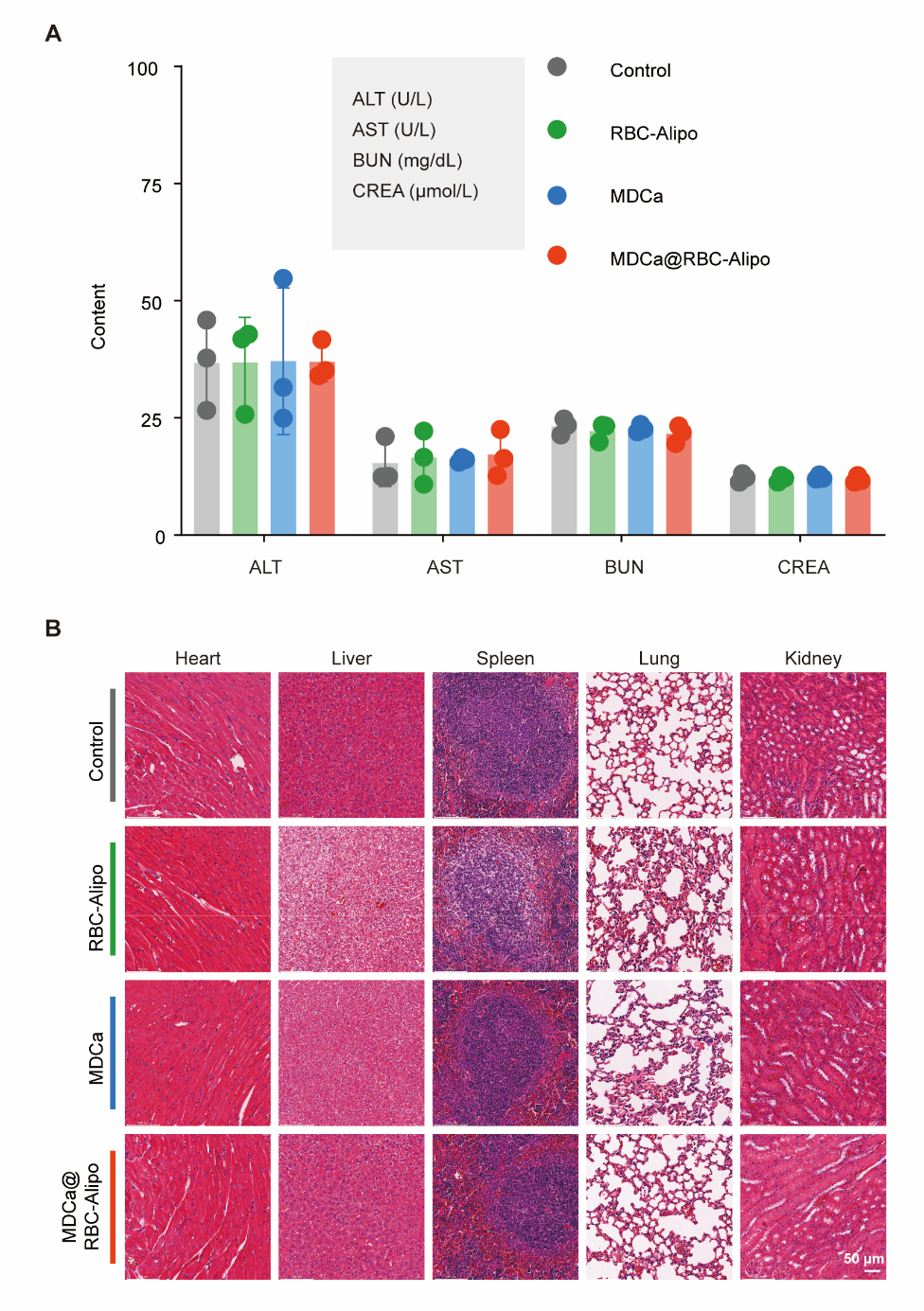


**Supplementary Figure 14**. **(A)** Serum biochemistry data including alanine aminotransferase (ALT), aspartate transaminase (AST), blood urea nitrogen (BUN), and creatinine (CREA) were measured after varied treatment. Data were expressed as mean ± SD (n=3); **(B)** H&E-stained tissue sections of major organs (liver, heart, spleen, lung and kidney) from mice with different treatments.


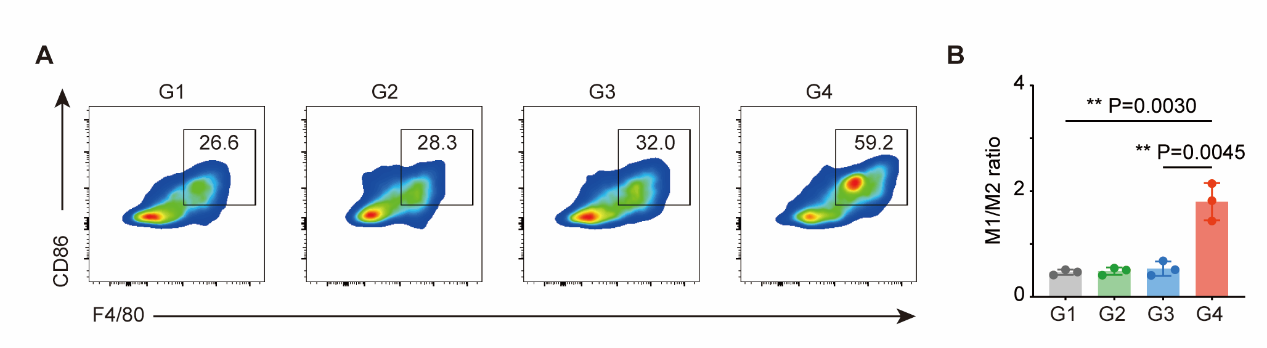


**Supplementary Figure 15**. **(A)** Representative flow cytometric analysis of TAM-M1 (CD86^hi^F4/80^+^CD11b^+^CD45^+^). **(B)** The ratio of M1/M2 in tumors after various kinds of treatment. Data were presented as means±SD(n=3). **P < 0.01.


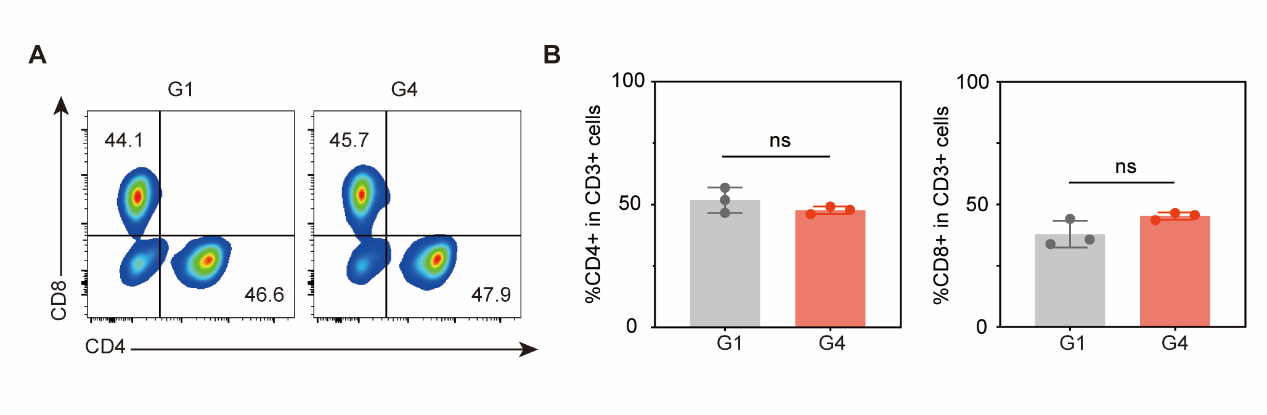


**Supplementary Figure 16**. Representative flow cytometric analysis **(A)** and relative quantification **(B)** of CD8^+^ T cells (CD8^+^CD3^+^CD45^+^) and CD4^+^ T cells (CD4^+^CD3^+^CD45^+^). Data were expressed as means ± SD(n=3). ns= not significant.


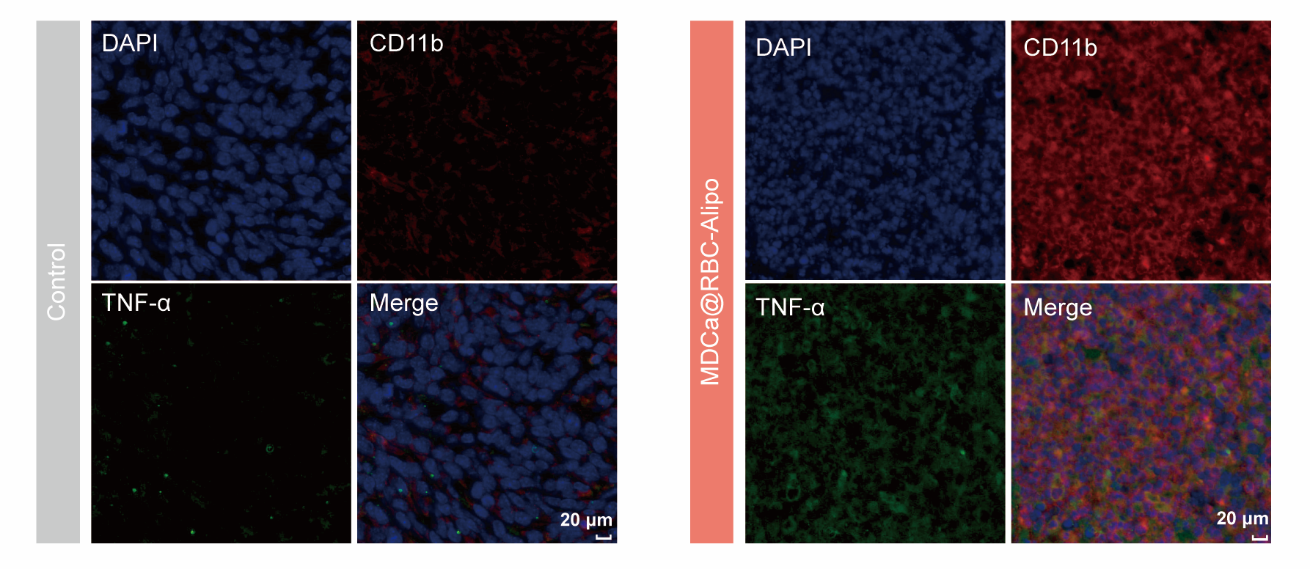


**Supplementary Figure 17.** CD11b^+^ myeloid cells within the tumor showed a significant increase in TNF-α production due to MDCa@RBC-Alipo treatment.


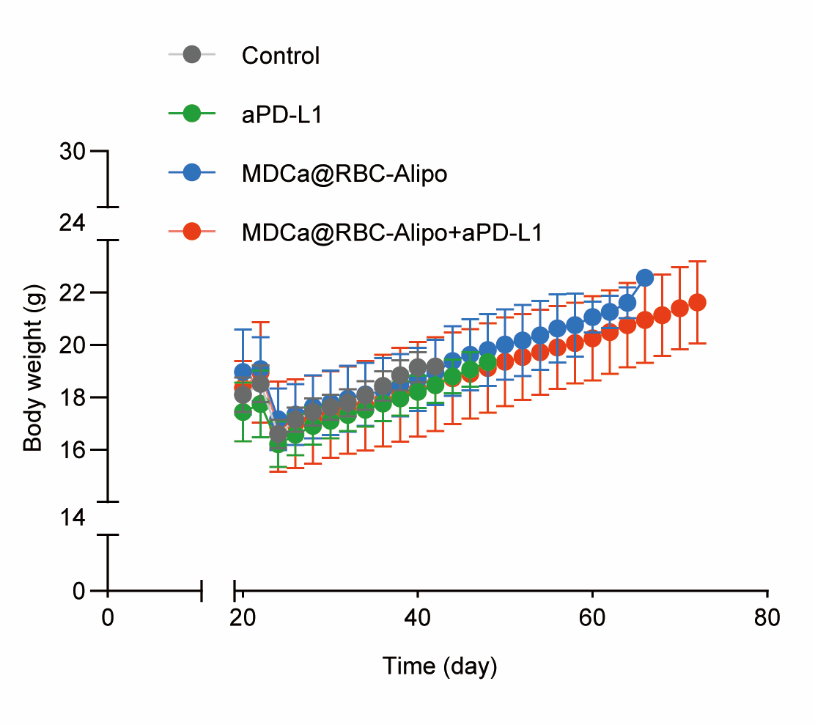


**Supplementary Figure 18**. Time-dependent body weight.


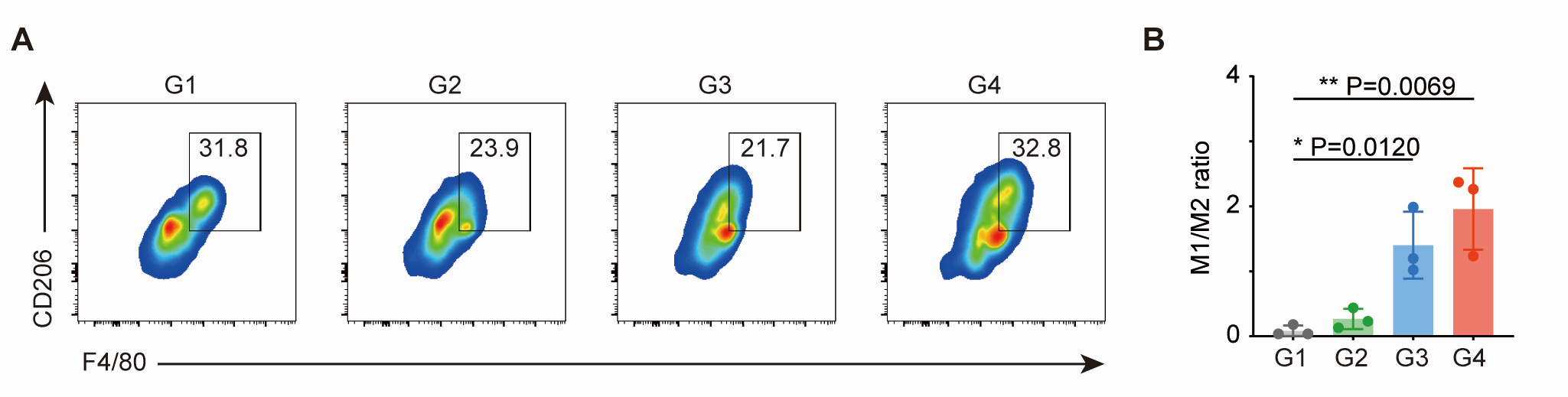


**Supplementary Figure 19**. **(A)** Representative flow cytometric analysis of TAM-M2 (CD206^hi^F4/80^+^CD11b^+^CD45^+^). **(B)** The ratio of M1/M2 in tumors after various kinds of treatment. Data were presented as means±SD(n=3). **P < 0.01 and ***P < 0.001.

.


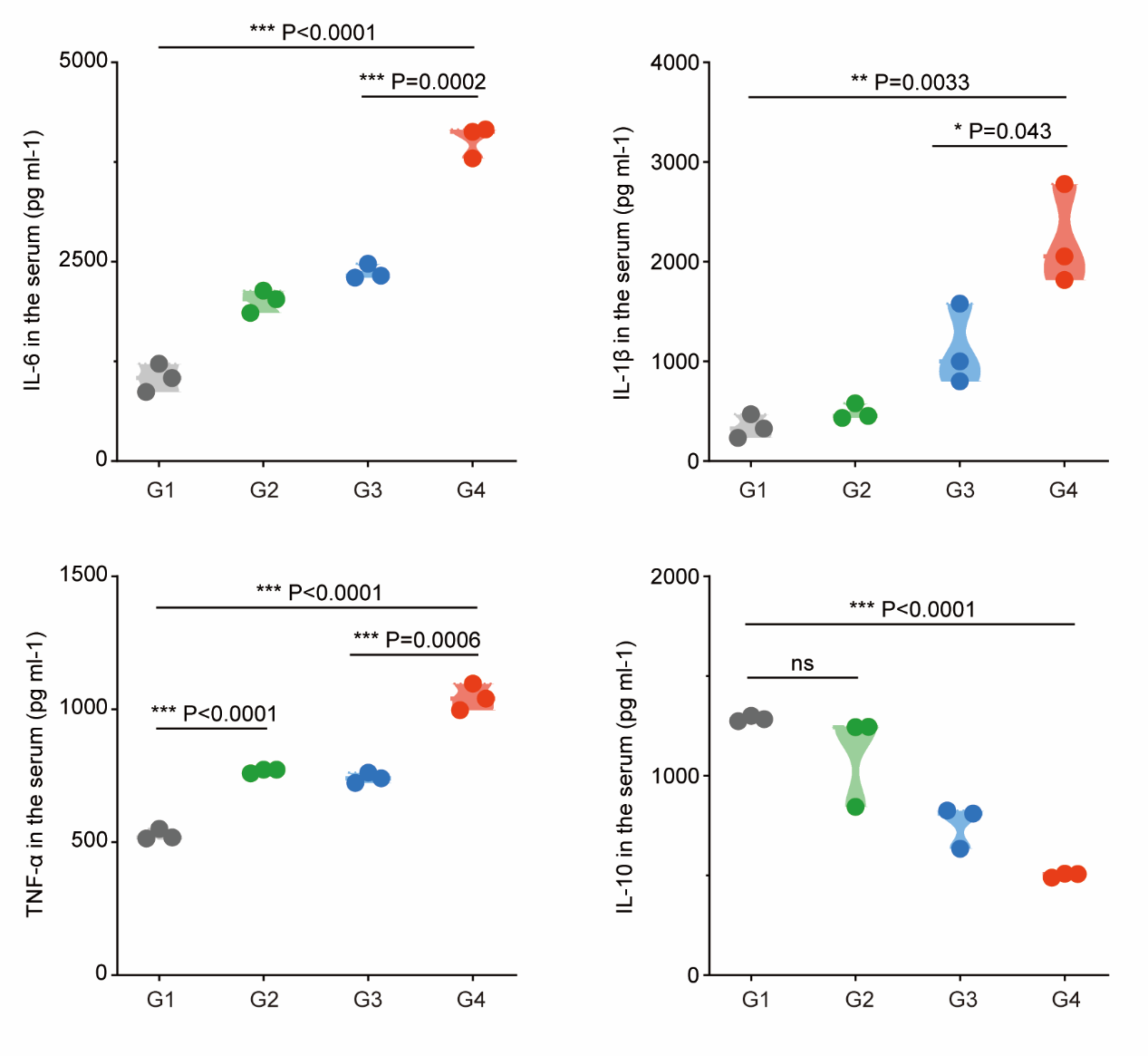


**Supplementary Figure 20**. Cytokine levels of TNF-α, IL-6, IL-1β, and IL-10 in the serum after various treatments. G1, Control; G2, aPD-L1; G3, MDCa@RBC-Alipo; G4, MDCa@RBC-Alipo+aPD-L1. Data were expressed as means ± SD (n=3). ns, not significant. *P < 0.05, **P < 0.01 and ***P < 0.001.


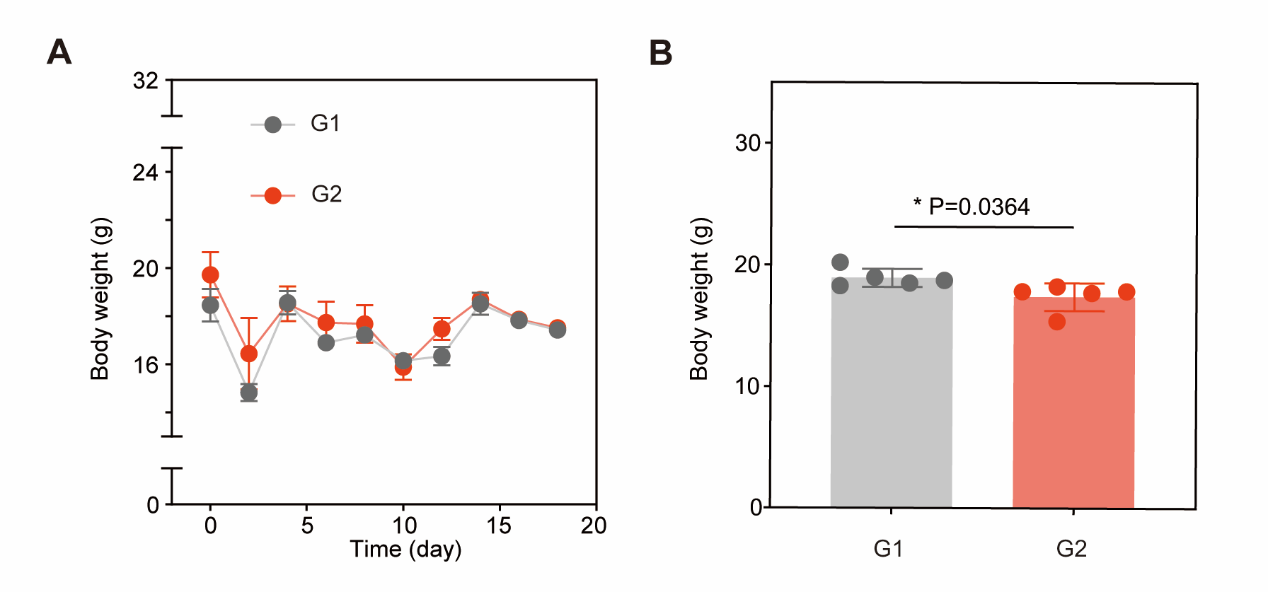


**Supplementary Figure 21**. Time-dependent body weight surveillance and the body weight at day18 after various treatments. G1:PBS, G2:MDCa@RBC-Alipo+aPD-L1. Data were expressed as means±SD (n=5). *P < 0.05.


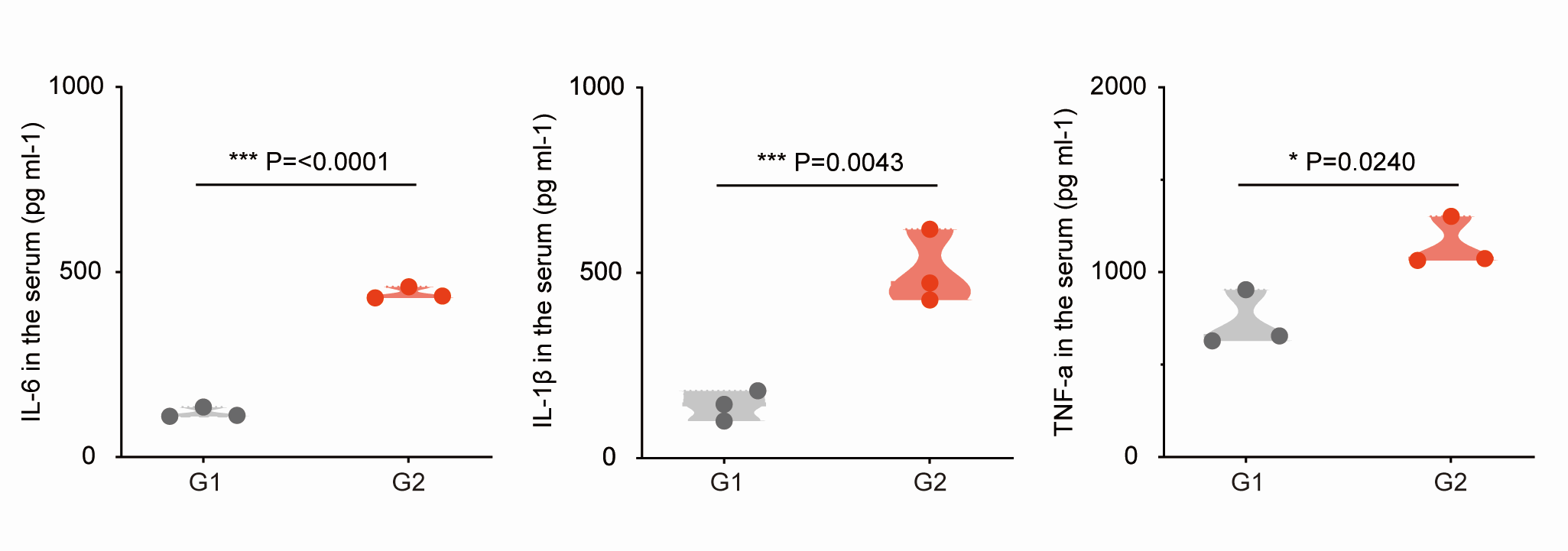


**Supplementary Figure 22**. Cytokine levels of IL-6, IL-1β and TNF-α in the serum after various treatments (n=3). G1:PBS, G2:MDCa@RBC-Alipo+aPD-L1. Data were expressed as means±SD. *P < 0.05 and ***P < 0.001.


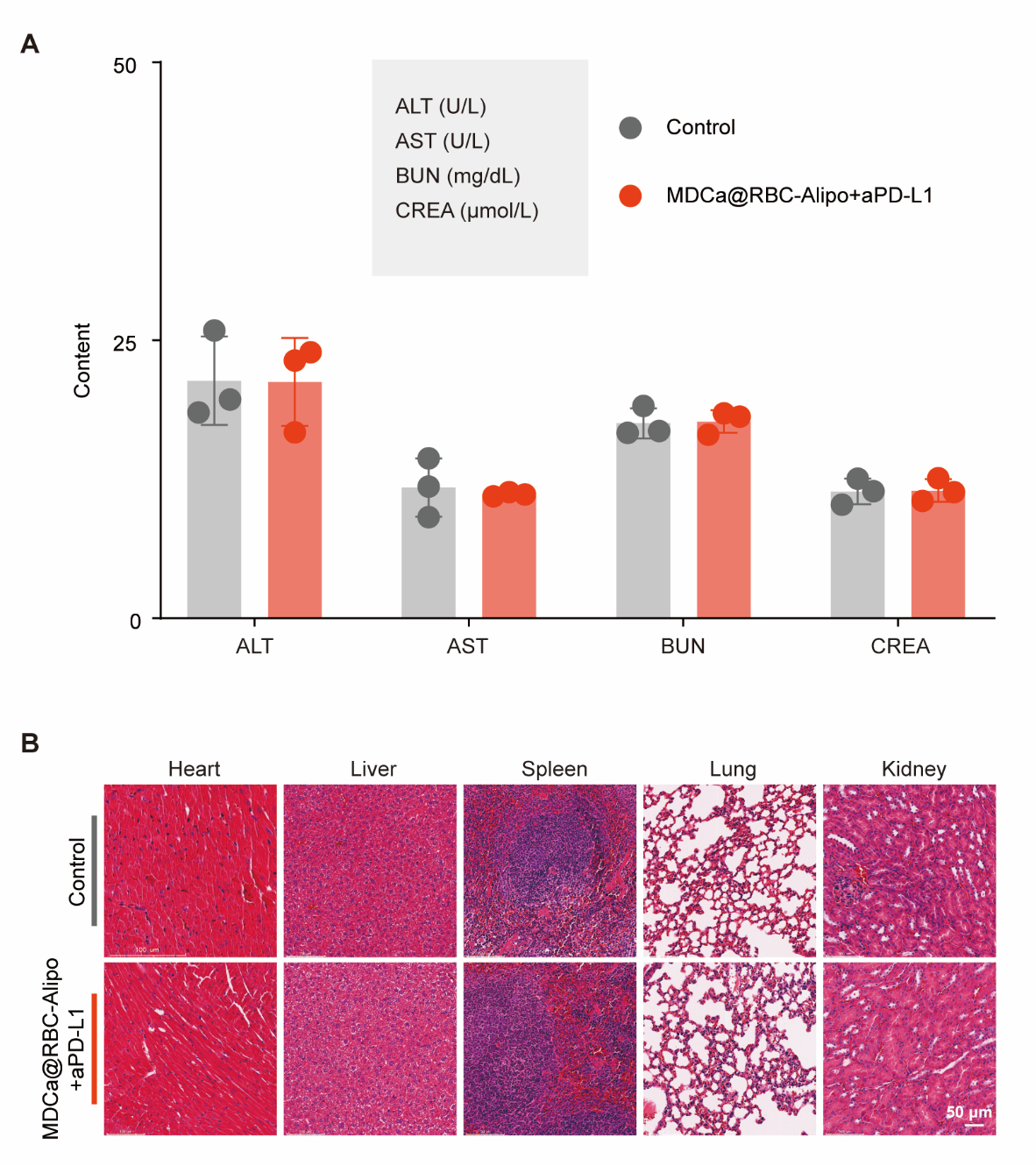


**Supplementary Figure 23**. **(A)** Serum biochemistry data including alanine aminotransferase (ALT), aspartate transaminase (AST), blood urea nitrogen (BUN), and creatinine (CREA) were measured after varied treatment. Data were expressed as mean ± SD (n=3) . **(B)** Representative H&E stained tissue sections of three biologically independent animals from each group.
